# Transferrin is a drug candidate for the treatment of dry age-related macular degeneration (AMD)

**DOI:** 10.1038/s41419-025-07950-0

**Published:** 2025-10-06

**Authors:** Jenny Youale, Karine Bigot, Thara Jaworski, Cécile Lebon, Anaïs Françon, Kimberley Delaunay, Romain Bénard, Thaïs De Bastard, Alejandra Daruich, Naël Kaddour, Thierry Bordet, Francine Behar-Cohen, Emilie Picard

**Affiliations:** 1 Centre de Recherche des Cordeliers, Inserm, Physiopathology of Ocular Diseases to Clinical Development, Université Paris Cité, Sorbonne Université, Paris, France; 2 PulseSight Therapeutics (formerly Eyevensys), Paris, France; 3 Ophthalmology Department, Necker-Enfants Malades University Hospital, AP-HP, Paris Cité University, Paris, France; 4 Cochin Hospital, AP-HP, Assistance Publique Hôpitaux de Paris, Paris, France

**Keywords:** Pathogenesis, Neurodegenerative diseases, Retina

## Abstract

Dysregulation of iron homeostasis plays a crucial role in retinal diseases, contributing to oxidative stress, inflammation, and ferroptosis, key processes that drive the degeneration of the retinal pigment epithelium (RPE) and photoreceptors in age-related macular degeneration (AMD). Previous studies, though limited in patient numbers, have reported elevated iron levels in the aqueous humor, RPE, and Bruch’s membrane of AMD patients. In this study, we aimed to confirm iron imbalance in a larger cohort of AMD patients and assess its correlation with disease stage. Elevated iron levels and a reduction in transferrin (TF) iron-binding capacity were observed in patients with early geographic atrophy (GA). RPE cells derived from human stem cells exhibited AMD-like features when exposed to iron overload or oxidized lipids. Treatment with TF appeared to restore aspects of iron homeostasis and reduce oxidative stress, mitochondrial damage, inflammation, complement activation, and ferroptosis in this model. These findings suggest that TF supplementation may represent a potential therapeutic strategy to help prevent or slow AMD progression.

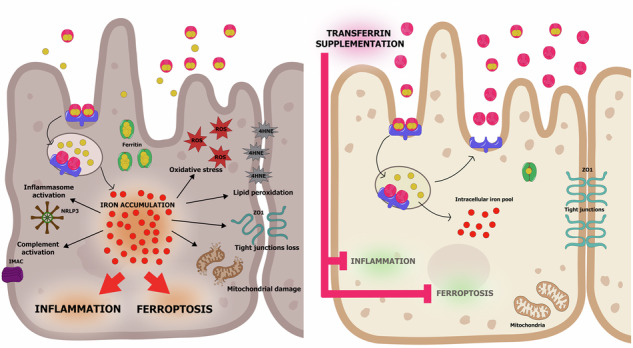

## Introduction

Iron is essential for retinal functions, but in excess, iron can be highly toxic. Proteins dedicated to iron transport and storage are produced locally in the eye, ensuring tight regulation of ocular iron homeostasis [1]. Free labile iron (Fe^2+^) is prone to generate highly reactive oxygen species via the Fenton reaction, which damage all the cellular components and ultimately cause cell death [2]. During aging, the iron that accumulates in the retina, particularly in the choroid, retinal pigment epithelium (RPE), and photoreceptors [3–5], together with reduced intracellular antioxidant defenses, predisposes to oxidative stress-induced cell death. Ferroptosis, a recently documented form of programmed cell death caused by iron-induced lipid peroxidation, has been implicated in the pathogenesis of several neurodegenerative retinal diseases [1], such as glaucoma [1, 6], inherited retinal dystrophies [7], and age-related macular degeneration (AMD) [8–10].

AMD is the leading cause of irreversible blindness in people aged 65 years and over in developed countries [1–4]. It is characterized by chronic dysfunction of the RPE, accumulation of drusen beneath the RPE, and progressive atrophy of the underlying choriocapillaris, which provides oxygen and nutrients to photoreceptor cells. In advanced AMD, choroidal neovascularisation leads to macular edema and bleeding (neovascular AMD), which can be alleviated by anti-vascular endothelial growth factor drugs [11]. In contrast, degeneration of RPE and outer retina results in geographic atrophy (GA), leading to progressive and irreversible vision loss for which treatment options remain very limited [12]. The first approved therapies for GA, complement C3 and C5 inhibitors, received FDA approval in early 2023 and represent a major milestone [13]. However, their modest efficacy in slowing GA lesion growth without demonstrating clear functional benefit, the burden of monthly or bi-monthly intravitreal injections, and potential safety concerns, including an increased risk of conversion to neovascular AMD, have limited their clinical use and precluded regulatory approval in Europe. These limitations underscore the urgent need for alternative or combination therapeutic strategies targeting distinct pathogenic mechanisms in GA. Oxidative damage and uncontrolled inflammation are major contributors to age-related RPE dysfunction, which threatens the integrity of the outer blood‒retinal barrier (oBRB) formed by the RPE cell layer [1, 14].

Iron accumulation and the dysregulation of proteins involved in iron homeostasis have been demonstrated in the retinas of AMD patients [15–20]. In vivo and in vitro models of retinal iron overload trigger the activation of the NOD-like receptor family pyrin domain-containing 3 (NLRP3) inflammasome signaling pathway [21] and the synthesis and cleavage of complement C3 [22], which activates the complement system pathway, two pathogenic mechanisms implicated in AMD [11, 23]. Interestingly, polymorphisms in genes encoding complement factors implicated in the risk of AMD and progression to GA do not necessarily influence the expansion of GA lesions and, in some cases, are even associated with slower lesion enlargement [24]. Mechanisms other than complement activation, such as ferroptosis, could be implicated in the expansion of RPE cell death in GA [9, 25–27]. In this context, strategies to neutralize iron represent new therapeutic options.

Transferrin (TF) is a key regulator of iron homeostasis in the retina and controls iron import into the eye. It also serves as a potent endogenous iron chelator, sequestering Fe^3+^ molecules with a very high affinity (10^22^M^−1^) at a 1:2 (TF:iron) ratio, preventing the accumulation of free radical-reactive labile iron in the retina. Under disease conditions, we previously demonstrated that TF protects retinal Müller glial cells, retinal ganglion cells, and photoreceptors from various forms of cell death, including apoptosis and necrosis [6, 28–30]. In multiple animal models of retinal degeneration, TF supplementation lowered retinal iron levels, reduced oxidative stress and inflammation, and preserved photoreceptor loss, leading to both anatomical and functional benefits [28–32]. Recently, we demonstrated that TF supplementation in a light-induced oxidative stress model also preserved the integrity of the RPE, suggesting that TF may help prevent RPE atrophy in GA [31].

To further explore the therapeutic potential of TF, we first aimed to confirm the imbalance in iron homeostasis among a larger cohort of dry AMD patients and assess its correlation with disease progression to GA. Additionally, we investigated the protective mechanisms of TF using human induced pluripotent stem cell (iPSC)-derived RPE (iRPE) cells exposed to iron overload or oxidized lipids.

## Materials and methods

### Iron status in ocular fluids

#### Patient selection and sample collection

Patients who underwent cataract surgery at Hospital Cochin (Paris, France) from September 1, 2017, to July 31, 2021, were included according to the protocol approved by the French local ethics Committee or “Comité de Protection des Personnes” Ile de France 1 (N°2016-nov-14390). All patients signed an informed consent form to be included in the study. The qualification of the stage of AMD was defined via the spectral-domain optical coherence tomography (SD-OCT) criteria established by the classification of atrophy meetings consensus (CAM) [33–35]. Incomplete RPE and outer retinal atrophy (iRORA) and RPE and outer retinal atrophy (cRORA) were used to classify patients according to the degree of atrophy. Patients assigned to the drusen group presented drusen without signs of atrophic lesions. The control group was assigned for patients presenting no ocular pathologies and underwent surgery for cataracts. Samples from patients with other ocular pathologies, such as diabetic retinopathy, AMD with choroidal neovascularization or fibrosis, high myopia, and uveitis, were discarded. Sex-matched and age-matched patients were included. Among the 135 aqueous humor (AH) samples selected, 77 samples were obtained from control patients (only cataracts), 58 from patients who presented with GA, 22 from patients who presented with drusen only, 17 from patients with iRORA, and 19 from patients with cRORA. The mean age of the control patients (40 males and 37 females) was 71.83 ± 7.4 years, and that of all the AMD patients (37 males and 21 females) was 77.48 ± 6.14 years. Specifically, the mean age for drusen (16 males and 6 females) was 77.91 ± 4.15 years; for iRORA (10 males and 7 females), it was 77.73 ± 7.39 years; and for cRORA (11 males and 8 females), it was 76.77 ± 7.05 years. For vitreous samples, 30 samples were selected (9 males and 21 females with a mean age of 71.21 ± 13.79 years) from patients who underwent surgery for macular holes or membrane peeling and who had no other ocular disease. Matched AH and vitreous samples were obtained from 6 control patients who underwent simultaneous cataract surgery and vitrectomy for membrane peeling.

Undiluted aqueous humor (at least 70 µl) and vitreous were sampled at the beginning of cataract surgery, collected in coded tubes, and then stored at −80 °C until analysis (DC-2016-2620).

#### Iron and transferrin assays

Aqueous humor and vitreous samples from humans were diluted with a 1% HNO_3_ solution containing 10 ng/mL Rh and 10 ng/mL indium as internal standards. In addition, each analytical batch of study samples was processed with laboratory controls, including method blanks and standard reference materials, to monitor method performance continuously. The samples were analyzed via an inductively coupled plasma mass spectrometer (ICP‒MS, 7700 Series, Agilent, Santa Clara, CA, USA). Quantitative analysis of iron (Fe) was carried out by external calibration using 7 standards, with concentrations ranging from 10 to 1000 ng/ml. The method was assessed via internal and external quality controls, initial calibration, a verification standard, procedural blanks, and duplicate samples.

Human transferrin was quantified via a custom-developed and validated electrochemiluminescence immunoassay (ECLIA). The assay utilized a goat anti-human TF antibody (Bio-Rad, Hercules, CA, USA) as the capture antibody, a goat biotinylated anti-human TF antibody (Bio-Rad) as the detection antibody, and sulfo-tagged streptavidin (MesoScale Diagnostics, Rockville, MD). The assay precision, accuracy, and linearity of dilution were evaluated, establishing a working range from 400 ng/mL to 20,000 ng/mL, which defines the lower and upper limits of quantification. The saturation of TF was calculated as the total iron content (µmol/l)/TIBC (µmol/l). TIBC measures the amount of iron that can potentially be stored in TF and corresponds to the capacity of 1 mol of TF (80,000 Da) to bind 2 mol of ferric iron and was calculated (µmol/l) as the TF concentration (g/l) × 10^6^ × (2/80,000).

### Cell culture

Human iPSC-derived RPE (iRPE) cells were obtained following the protocol described by Udry et al. [36] and characterized in our previous paper [37, 38]. Briefly, following the differentiation of hiPSCs into iRPE cells, iRPE cells were seeded at passages 3 or 4 in Transwell inserts (12 or 24 mm with a 0.4 μm pore polyester membrane insert; Corning, Manassas, VA, USA) on a Matrigel coating (Corning) for 42–72 days in serum- and antibiotic-free retinal differentiation medium containing 40% DMEM (high glucose, GlutaMAX Supplement, HEPES; Thermo Scientific), 20% Ham’s F-12 Nutrient Mix (Thermo Scientific) and 2% B-27 supplement minus vitamin A (Thermo Scientific). One week before stress induction, the iRPE cell medium was replaced with red phenol-free DMEM (high glucose, HEPES; Thermo Scientific) supplemented with 10% FBS charcoal stripped for transepithelial resistance stability. Stress induction was performed in serum-free DMEM. All the cultures were maintained at 37 °C in a humidified incubator with 5% CO_2_/95% air. Three independent pools of cells were used for this study. The assignment of cell wells was done by blind operator.

### Toxicological screening

iRPE cells were exposed to increasing concentrations of FeCl_3_-nitrilotriacetate (FeCl_3_NTA; Merck, Saint Quentin Fallavier, France) and 4-hydroxy-2-nonenal (4HNE; Merck) in the medium for 24 h. 4-hydroxy-2-nonenal (4HNE), a major product of lipid peroxidation, increased in aged human retina [39] and induces an AMD phenotype-like in RPE cells [40]. The cells were also exposed to human apo-TF (TF; Merck) in the absence of a stress inducer to document its safety profile. Untreated cells were used as controls.

### Stress induction

iRPE cells were exposed to FeCl_3_NTA (1 mM) or 4HNE (120 µM) for 24 h, and TF (10–20 mg/mL) was co-administered with the stress inducer. For sustained iron overload conditions, TF (10 mg/mL) was added to the culture after 24 h of iron exposure (0.5 mM FeCl_3_NTA). Untreated cells were used as controls.

### Cell viability analysis

Cell viability was determined via the colorimetric method CellTiter 96® Aqueous (Promega Corporation, Charbonnières-Les-Bains, France), which measures mitochondrial activity according to the manufacturer’s instructions. The results are expressed as a percentage of the colorimetric measurement of untreated-cell viability. For oxidative stress conditions, lactate dehydrogenase (LDH) release in the cell culture medium was also measured via a Cytotoxicity Detection Kit reaction mixture (Roche Diagnostics, Meylan, France) according to the manufacturer’s instructions. The results are expressed as the percentage of total LDH released by iRPE cells. Untreated cells were used as controls.

### Transepithelial resistance

Transepithelial resistance (TEER, Ω.cm²) measurements were performed via an EVOM2 Epithelial Voltohmmeter with an STX2 electrode (World Precision Instruments, Sarasota, Florida, www.wpiinc.com). Each value was determined from the average of multiple independent wells and corrected for background resistance produced by a blank filter with stress culture medium. Untreated cells were used as controls.

### H_2_O_2_ detection

The release of H_2_O_2_, a reactive oxygen species produced by iRPE cells, was detected via a bioluminescence assay via a ROS-Glo™ kit (Promega, Charbonnières-les-Bains, France) according to the manufacturer’s instructions. Stress inducer-untreated cells were used as controls. The values are reported as the average bioluminescence signal of the control condition.

### Perls’ Prussian blue staining

The Perls’ Prussian blue stain consists of iron detection by 2% ferrocyanide in 2% aqueous hydrochloric acid. The reaction was enhanced by incubation in 0.05% 3,3-diamino-benzidine and 0.01% H_2_O_2_ in a Tris-buffered saline solution. Pictures were taken using a Leitz microscope and photographed with a Leica camera. The gray level was quantified in each condition and expressed by subtracting the baseline gray level obtained from control condition with ImageJ software.

### Immunocytochemistry

iRPE cells were fixed in 4% paraformaldehyde (PAF, Inland Europe, Conflans sur Lanterne, France) for 20 min. Flat-mounted Transwells or cryosections of snap-frozen iRPE cells in OCT (Tissue Tek, Siemens Medical, Puteaux, France) were permeabilized and blocked respectively in 1% Triton X-100 (Merck) in PBS and 1% bovine serum albumin (BSA; Merck)/0.1% Triton in PBS, followed by overnight incubation with goat anti-rabbit ZO1 (1:200; 40-2200, Invitrogen, France), goat anti-mouse NLRP3 (1:100; ALX-804-819, Enzo Life Sciences, Farmingdale, NY, USA), goat anti-mouse C5b9 (1:50; sc-58935, Santa Cruz Biotechnologies, Heidelberg, Germany), goat anti-mouse AIF1 (1:50; sc-13116, Santa Cruz Biotechnologies), goat anti-rabbit 4HNE (1:100; ab46545, Abcam, Cambridge, UK) and goat anti-rabbit NRF2 (1:100; sc-722, Santa Cruz Biotechnologies) primary antibodies. The corresponding Alexa Fluor-conjugated donkey anti-rabbit (A10040), chicken anti-rabbit (A21441), or donkey anti-mouse (A21202) IgG secondary antibodies (1:200; Thermo Fisher Scientific) were used as primary antibodies, and the nuclei of the cells were counterstained with 4.6-diamidino-2-phenylindole (DAPI; Merck). The primary and secondary antibodies were diluted in 0.5% BSA/0.1% Triton in PBS. Pictures were acquired with a fluorescence microscope (BX51, Olympus; Rungis, France, or Confocal Zeiss LSM 710; Zeiss, Germany) using identical exposure parameters for all compared samples. For NLRP3, AIF1, and nuclear NRF2 immunofluorescence labeling, the results are expressed as the fold change versus the baseline fluorescence intensity quantified in control cells. For zonula occludens 1 (ZO1) immunofluorescence labeling, the results are expressed as the fold change versus the baseline fluorescence intensity quantified in control cells, and the number of ZO1 fragments was counted to evaluate the discontinuity of the tight junctions. For 4HNE and C5b9 immunofluorescence labeling, the number of particles per area was quantified. All analyses were performed with ImageJ software (v1.52p).

### Western blot

Proteins were extracted with M-PER™ Mammalian Protein Extraction Reagent (Thermo Fisher Scientific), and their concentrations were determined via the Micro BCA protein assay (Thermo Fisher Scientific). Twenty micrograms of protein extract were run on a 4–12% Bis‒Tris gel (Thermo Fisher Scientific) and transferred to a nitrocellulose membrane. The blots were blocked 1 h in 5% nonfat dry milk in Tris-buffered saline solution at room temperature. The membranes were subsequently incubated overnight at 4 °C with goat anti-rabbit FTL (1:1,000; Homemade gift from P. Santambrogio), goat anti-rabbit FPN (1:1,000; A14884, ABclonal, Dusseldorf, Germany), goat anti-rabbit GPX4 (1:5000; ab125066, Abcam), and goat anti-rabbit TFR1 (1:500; A5865, ABclonal) primary antibodies in 5% nonfat dry milk in 0.05% Tween/Tris-buffered saline solution. Then, the blots were incubated with the corresponding HRP-conjugated secondary antibody (dilution 1:5000; P1-1000, Eurobio Scientific) for 1 h at room temperature. The proteins were detected with SuperSignal West Pico PLUS chemiluminescence substrate (Thermo Fisher Scientific) and visualized with an iBright Western Blot Imaging System (Thermo Fisher Scientific). The gray values of specific bands were analyzed via ImageJ software (v1.52p). The signal of the proteins of interest was reported relative to the signal from the total proteins labeled with the Pierce Reversible Membrane Stain™ Kit (Thermo Fisher Scientific). The results are expressed as the fold change versus the baseline gray intensity quantified in control cells. The unprocessed original images of Western blot results were provided in the Additional File 1.

### RT-qPCR

Total RNA was extracted from iRPE cells via a RNeasy Mini Kit (Qiagen, Courtaboeuf, France) according to the manufacturer’s protocols. After assessment of its purity and integrity via a Nanophotometer (Implen, München, Germany) and evaluation of its concentration, RNA was reverse transcribed into cDNA with GoScript Reverse Transcription Mix, Oligo(dT) (Promega). Quantitative PCR was performed via the use of primers for the human target genes involved in iron homeostasis, ferroptosis, and inflammation processes (Supplementary Tables 1 and 2) with GoTaq qPCR Master Mix (Promega) via a Quant Studio 5 PCR System (Thermo Fisher Scientific). All samples were run in duplicate, and the fluorescent threshold values (Ct) were determined using the Quant Studio Design and Analysis software (v1.4; Thermo Fisher Scientific). The expression level of individual genes was normalized to that of the housekeeping gene *RPLP0* (NM_001002; QT00075012, Qiagen) in the same sample by calculation of the ΔCt value, and relative quantification was calculated via the ΔΔCt method with control cells.

### Statistical analysis

The results are presented as the mean ± standard deviation (SD) or standard error of the mean (SEM). Analyses were performed using GraphPad Prism 8 software, and the nonparametric Kruskal‒Wallis test and Dunn’s test were used to compare groups with fewer than five samples (n < 5). For groups equal to or greater than five samples (n ≥ 5), the Shapiro‒Wilk W test was used to evaluate each group for normality. Parametric ANOVA and the Bonferroni correction were used if normality was validated; otherwise, the nonparametric Kruskal‒Wallis’s test and Dunn’s test were used to compare groups. Significant results have a *p* value less than 0.05.

## Results

### Iron-TF imbalance in the aqueous humor of patients with AMD

**Fig. 1:**
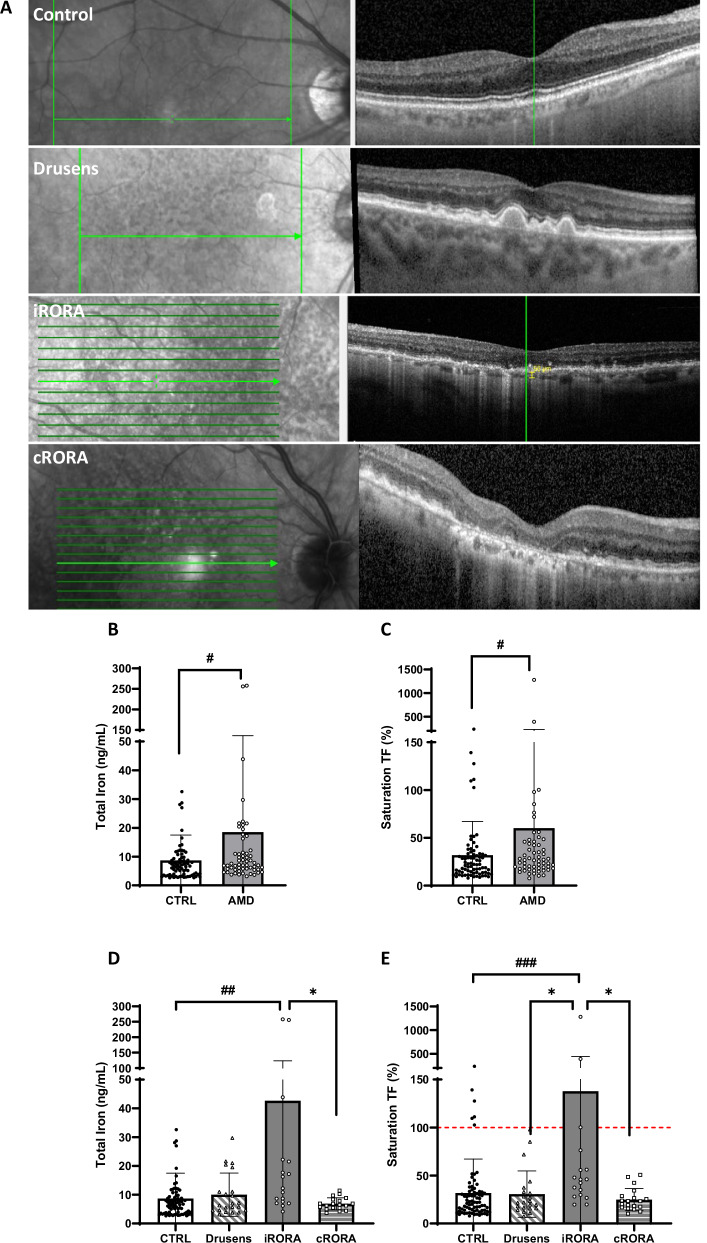
Atrophic geography age-related macular degeneration (AMD) patients present a high transferrin saturation. **A** Representative images of spectral-domain optical coherence tomography (SD-OCT) in a healthy eye from a control patient and in eyes from patients presenting drusen only, incomplete retinal pigment epithelium (RPE) and outer retinal atrophy (iRORA), and complete RPE and outer retinal atrophy (cRORA). Significant differences in the concentrations of iron (**B**) and transferrin (TF) saturation (**C**) in the aqueous humor between the control eyes (*n* = 77) and all AMD eyes (*n* = 58). Bars represented means ± SD. Mann-Whitney test; *p*# < 0.05. The concentrations of iron (**D**) and TF saturation (**E**) were significantly higher in iRORA patients than in control patients, drusen, and cRORA patients. Bars represented means ± SD. Kruskal–Wallis test; Dunn’s post-hoc test; *p*# < 0.05, ##*p* < 0.01, ####*p* < 0.0001 compared with the control; *p < 0.05 compared with the AMD groups.

Patients with AMD had significantly higher levels of total iron in the aqueous humor (AH) than did age-matched patients without AMD (median: 6.665 vs. 7.103; interquartile range: 3.62–9.52 vs. 5.49–11.6; *p* = 0.024; Fig. 1B). While the concentration of transferrin (TF) was not significantly different between the control and AMD patients (Supplementary Fig. 1A), the saturation of TF, which corresponds to the percentage of iron-binding sites of TF occupied by iron, reached 59.5% in AMD patients compared with 31.8% in control patients (*p* = 0.036, Fig. 1C). Subgroup analyses stratified by sex revealed no statistically significant differences in total iron levels, TF concentrations, or TF saturation in between males and females within either the control or AMD groups, although slightly higher iron levels were observed in male AMD patients (Supplementary Fig. 2A–C). Given the lack of significant sex-based differences and the limited sample sizes within subgroups, all comparisons were performed using disease status as the primary variable to preserve statistical power and maintain analytical robustness.

The AMD patients were divided following the spectral-domain optical coherence tomography (SD-OCT) criteria established by the classification of atrophy meetings consensus (CAM) [33, 34] (Fig. 1A). Among AMD subtypes, total iron levels were significantly higher in incomplete retinal pigment epithelium (RPE) and outer retinal atrophy (iRORA) patients compared to control patients (*p* = 0.0013), whereas no differences were detected in patients with drusen only (without any signs of atrophy) or complete RPE and outer retinal atrophy (cRORA) compared with controls (Fig. 1D). The TF concentration remained similar across AMD subtypes and the control group (Supplementary Fig. 1B). Thus, TF saturation in iRORA patients was significantly greater than that in controls, reaching 138% (*p* = 0.0005; Fig. 1E). TF saturation was also significantly higher in the iRORA group than in the drusen and cRORA groups (*p* = 0.021 and *p* = 0.015, respectively; Fig. 1E).

To estimate the value of measurements taken in the AH compared with those present in the vitreous, which is closer to the retina, we quantified iron and TF in 30 vitreous samples from control individuals. Interestingly, the level of iron in the vitreous was almost 12 times higher than that in the AH (*p* < 0.0001), with no difference in TF concentration, leading to a TF saturation reaching 98.8% in the vitreous of the controls (*p* < 0.0001) compared with 31.7% in the AH of the controls (Supplementary Fig. 3A, B). Higher iron level and TF saturation were also observed in the AH and vitreous simultaneously collected from 6 control patients (Supplementary Fig. 3C, D).

Taken together, these results not only confirm previous observations of iron accumulation in AMD eyes but also reveal an iron-transferrin imbalance, with the most pronounced effects observed in nascent GA (iRORA).

### TF protects human RPE cells from 4HNE-induced features resembling AMD

**Fig. 2:**
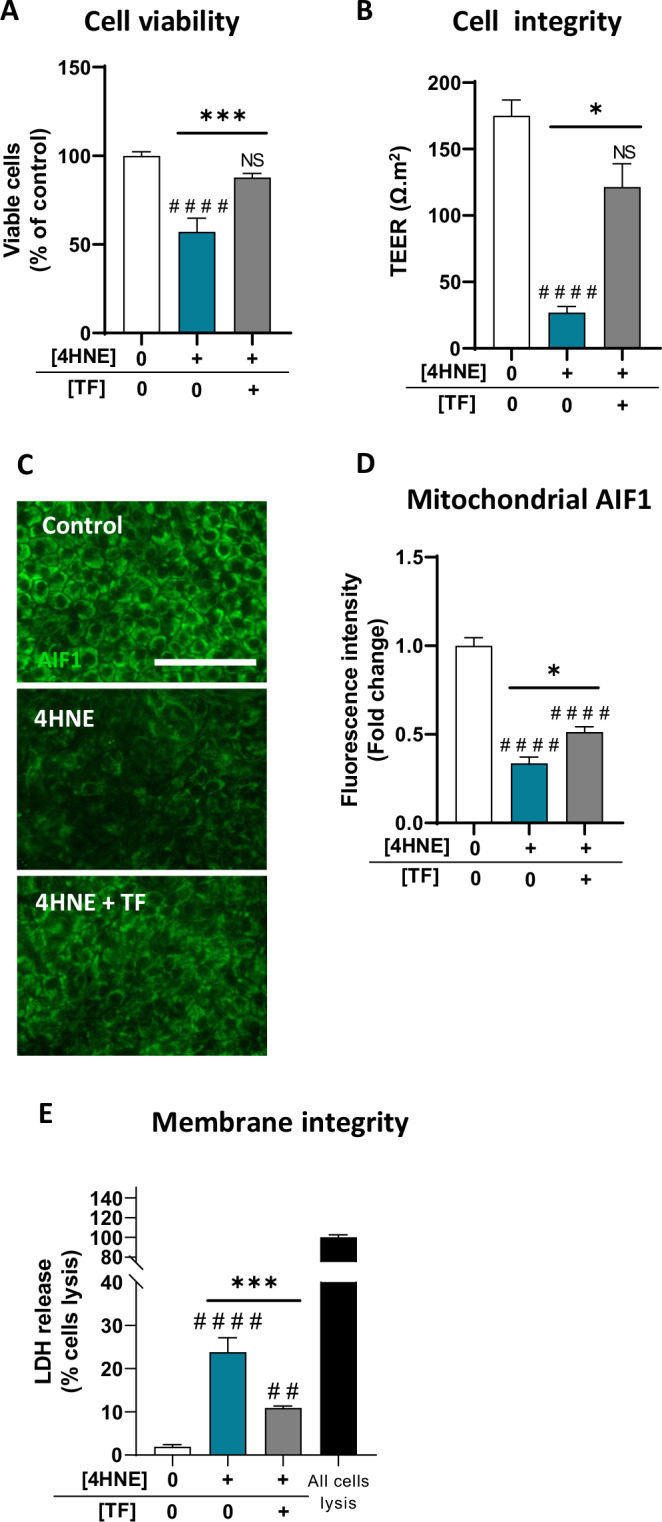
Transferrin preserved iRPE cells from 4HNE-induced toxicity. **A** A 24-h incubation with 120 µM 4-hydroxy-2-nonenal (4HNE) significantly reduced human induced pluripotent stem cell (iPSC)-derived RPE (iRPE) cell viability compared with that of the control. Co-treatment with 10 mg/mL TF significantly protected iRPE cells from 4HNE-induced toxicity (*n* = 8 wells per condition). **B** Transepithelial electrical resistance (TEER) was measured to evaluate iRPE cell integrity. TF co-treatment significantly prevented the decrease in TEER caused by 4HNE stress (*n* = 11–12 wells per condition). **C** Apoptotic-induced factor 1 (AIF1) immunofluorescence-labeled mitochondria in iRPE cells treated with 4HNE or co-treated with TF (scale bar: 100 µm) were quantified (**D**), revealing the preservation of AIF1 staining when TF was added to 4HNE (*n* = 4 wells per condition). **E** Under 4HNE stress, membrane integrity, monitored by the release of lactate dehydrogenase (LDH), was lost, and TF co-treatment significantly prevented this loss (*n* = 8 wells per condition). Untreated cells were used as control. Bars were means ± SEM. One-way ANOVA with post-hoc Bonferroni test (**A**, **E**) or Kruskal‒Wallis test with Dunn’s post-hoc test (**B**, **D**); NS non-significant ##*p* < 0.01, ####*p* < 0.0001 compared with the control; *p < 0.05, ***p < 0.001 compared with the 4HNE stress.

**Fig. 3:**
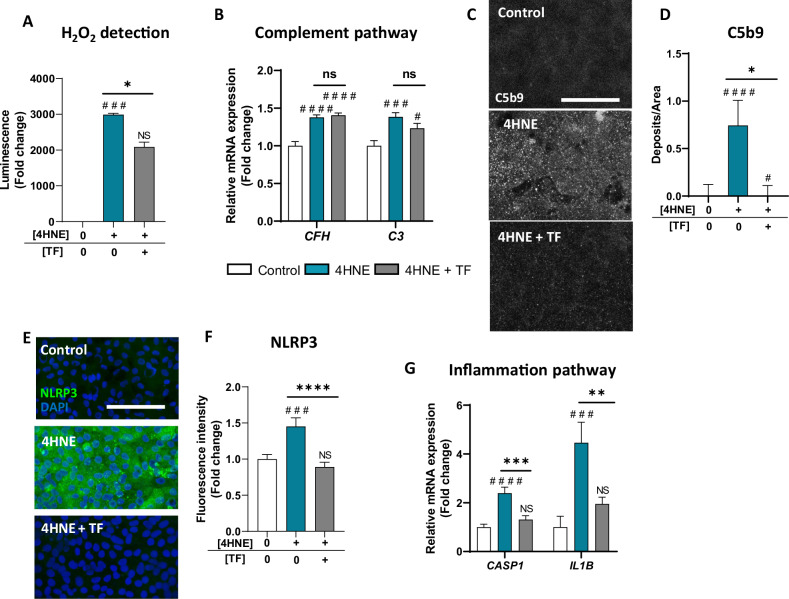
4HNE mimics AMD features, and TF provides protection. **A** H_2_O_2_ released during 4HNE stress (120 µM) was decreased by TF co-treatment (10 mg/mL) (*n* = 5–6 wells per condition). **B** Complement 3 (*C3*) and complement factor H (*CFH*) gene expression in iRPE cells, as determined by RT‒qPCR, was increased by 4HNE, and TF co-treatment prevented *C3* upregulation (*n* = 6 wells per condition). C5b9 membrane attack complex immunofluorescence labeling (**C**) was used to quantify the number of C5b9 deposits (**D**), revealing an increase under 4HNE stress condition, which was limited by TF co-treatment (**C**: scale bar: 100 µm; **D**: n = 4 wells per condition). The inflammasome revealed by NOD-like receptor family pyrin domain-containing (NLRP3) immunofluorescence labeling in iRPE cells (**E**) was quantified (**F**) and showed increased fluorescence intensity with 4HNE stress, which was prevented by TF co-treatment (**E**: scale bar 100 µm; **F**: n = 8 wells per condition). **G** 4HNE stress significantly increased interleukin-1 beta (*IL1B*) and caspase 1 (*CAS1*) gene expression, as determined by RT‒qPCR. TF co-treatment maintained their expression at the control level (*n* = 6 wells per condition). Untreated cells were used as control. Bars were means ± SEM. One-way ANOVA with post-hoc Bonferroni test (**B**, **G**) or Kruskal‒Wallis test with Dunn’s post-hoc test (**A**, **D**, **F**); NS non-significant, #*p* < 0.05, ###*p* < 0.001, ####*p* < 0.0001 compared with the control; ns non-significant, *p < 0.05, **p < 0.01, ****p < 0.0001 compared with the 4HNE stress.

Given the increased TF saturation rate in AMD, especially in iRORA eyes, we investigated the protective effects of TF supplementation in an in vitro model of oxidative stress-induced RPE dysfunction. First, human RPE cells differentiated from hiPSCs (iRPE) were exposed to increasing concentrations of TF for 24 h, and TF had no effect on cell viability or integrity at concentrations up to 20 mg/mL (Supplementary Fig. 4A, B). Then, iRPE were exposed to 4-hydroxy-2-nonenal (4HNE), a major product of lipid peroxidation that induces mitochondrial damage, membrane integrity disruption, inflammatory processes, and lipid peroxidation-induced ferroptosis, all of which are related to GA features [40]. We chose to use 4HNE to study the consequence of lipid peroxidation on RPE because it has been shown to accumulate with aging in the human retina [41], 4HNE-modified proteins have been identified in the retina of eyes with AMD [42], and in human RPE cells [43]. Moreover, 4HNE is produced in RPE cells exposed to cigarette smoke in vitro, which is the strongest environmental risk factor for AMD [44]. In addition, 4HNE has been detected in drusen in the monkey eye, which develops features similar to AMD in humans [45]. At concentrations greater than 120 µM, compared with the control, 4HNE significantly reduced iRPE cell viability (by ~50%, *p* < 0.0001) and disrupted cell integrity, as measured by an 84.6% reduction in transepithelial resistance (TEER) (*p* < 0.0001; Supplementary Fig. 4C, D). Co-incubation with TF (10 mg/mL) efficiently preserved both iRPE cell viability and cell integrity from 4HNE toxicity (Fig. 2A, B). Similarly, TF protected differentiated ARPE-19 from 4HNE toxicity (Supplementary Fig. 5A, B). Immunofluorescence of the mitochondrial protein Apoptotic-induced factor 1 (AIF1), indicative of mitochondrial health, revealed reduced staining under 4HNE exposure (−66.5% compared with the control, *p* < 0.0001), whereas co-incubation with TF significantly increased AIF1 staining by 34.6% compared with 4HNE alone (*p* = 0.0369; Fig. 2C, D). The disruption of membrane integrity quantified by lactate dehydrogenase (LDH) release in cells exposed to 4HNE was significantly reduced by TF (*p* < 0.0001; Fig. 2E). As expected, 4HNE-induced oxidative stress and pro-inflammatory pathways in iRPE cells (Fig. 3). The drastic increase in H_2_O_2_ release (*p* = 0.0002) was significantly reduced by TF (*p* = 0.0396, Fig. 3A). The increase in complement C3 (*p* = 0.0007) and complement factor H (*CFH*, *p* < 0.0001) gene expression and the accumulation of complex attack membrane C5b9 deposits on iRPE cells (*p* < 0.0001; Fig. 3B–D) were all induced by 4HNE. TF prevented the upregulation of C3 gene expression (*p* = 0.0007 versus *p* = 0.0215 when compared to control level) and the accumulation of C5b9 membrane deposits (*p* = 0.0469 compared to 4HNE condition), but it did not reduce *CFH* gene expression (Fig. 3B–D). Furthermore, 4HNE triggered inflammasome activation, as evidenced by increased NLRP3 immunofluorescence intensity (*p* = 0.0009; Fig. 3E, F) and the upregulation of caspase 1 (*CASP1*) and interleukin-1 beta (*IL1B*) gene expression (*p* < 0.0001 and *p* = 0.0007, respectively; Fig. 3G). TF prevented inflammasome pathway activation, restored NLRP3 fluorescence intensity, and normalized *CASP1* and *IL1B* gene expression to control levels (Fig. 3E–G). TF prevented the activation of both oxidative and inflammatory pathways in iRPE cells exposed to 4HNE.

**Fig. 4:**
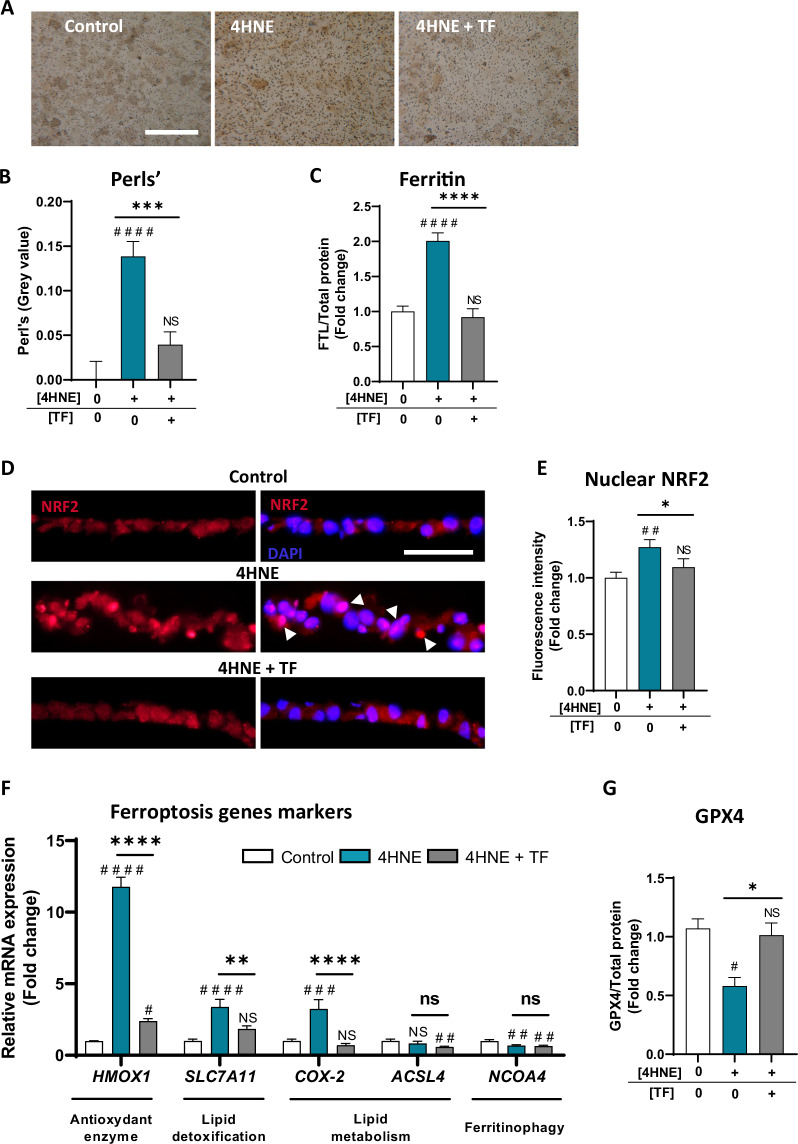
Transferrin preserved iRPE cells from 4HNE-induced ferroptosis through iron import and export. Iron deposits were revealed by 3,3′-diaminobenzidine (DAB)-amplified Perl’s reaction (**A**) in iRPE cells, and the staining intensity was evaluated (**B**). The iRPE cells treated with 120 µM 4HNE showed intracellular iron accumulation, which was not observed when TF (10 mg/mL) was added (**A**: scale bar 100 µm; **B**: n = 4 wells per condition). **C** The protein expression of ferritin light chain (FTL) increased under 4HNE condition, was maintained at the control level with TF co-treatment (*n* = 5–6 wells per condition). Nuclear factor erythroid-2-related factor (NRF2) immunofluorescence staining of cryosections of iRPE cells (**D**) was quantified (**E**) and showed a nuclear translocation of NFR2 with 4HNE, which was lower with TF co-treatment (**D**: scale bar 50 µm; **E**: n = 4 wells per condition). **F** The gene expression of the ferroptosis markers heme oxygenase 1 (*HMOX1*), solute carrier family 7 member 1 (*SLC7A11*), cyclooxygenase 2 (*COX-2*), acyl-CoA synthetase long chain family member 4 *(ACSL4*), and nuclear receptor coactivator 4 (*NCOA4*) was evaluated via RT‒qPCR. TF co-treatment significantly prevented the increase in *HMOX1*, *SLC7A11*, and *COX-2* gene expression caused by 4HNE intoxication (*n* = 6 wells per condition). **G** The glutathione peroxidase 4 (GPX4) protein level quantified by Western blot decreased after 24 h of 4HNE stress but was preserved to the control level by TF treatment (*n* = 4 wells per condition). Untreated cells were used as control. Bars were means ± SEM. One -way ANOVA with post- hoc Bonferroni test (**C**, **F**) or Kruskal‒Wallis test with Dunn’s post-hoc test (**B**, **E**, **G**); NS non-significant, #*p* < 0.05, ##*p* < 0.01, ###*p* < 0.001, ####*p* < 0.0001 compared with the control; ns non-significant, *p < 0.05, **p < 0.01, ***p < 0.001, ****p < 0.0001 compared with the 4HNE stress.

We then evaluated the capacity of TF to protect against ferroptotic cell death, which is characterized by intracellular iron accumulation and an imbalance of the antioxidant system. Exposure to 4HNE caused iron accumulation in iRPE cells (*p* < 0.0001; Fig. 4A, B) with increasing expression of the iron storage protein ferritin light chain (FTL, *p* < 0.0001; Fig. 4C). TF co-incubation significantly reduced intracellular iron accumulation (−71.5% compared to stress condition, *p* = 0.0007; Fig. 4A, B), as confirmed by the fact that FTL protein expression was maintained at control levels (*p* < 0.0001 compared with that under stress conditions) (Fig. 4C). The antioxidant system involved in the defense against ferroptosis is mainly controlled by the transcription factor nuclear factor erythroid-2-related factor (NRF2), which is known to induce the expression of antioxidant enzymes and lipid detoxification enzymes [46]. Under stress conditions, sections of iRPE cells showed nuclear translocation of NRF2 (*p* = 0.0350; Fig. 4D, E). NRF2 signaling activity was confirmed by the increase in the expression of genes encoding targets involved in ferroptosis, such as heme oxygenase 1 (*HMOX1*, *p* < 0.0001) and solute carrier family 7 member 1 (*SLC7A11*, *p* < 0.0001) (Fig. 4F). In the presence of TF, NRF2 nuclear translocation was maintained at control levels (*p* < 0.05 compared with the stress condition; Fig. 4D, E), and *HMOX1* and *SLC7A11* gene expression was significantly reduced (*p* < 0.0001 compared with that under stress; Fig. 4F). Despite NRF2 activity, 4HNE intoxication induced a significant increase in the gene expression of cyclooxygenase 2 (*COX-2*/*PTGS2*, *p* = 0.0005 compared with the control; Fig. 4F), which is known to promote the formation of proferroptotic polyunsaturated fatty acids (PUFA). Ferroptosis was confirmed by a decrease in the lipid detoxification system, such as glutathione peroxidase 4 (GPX4, *p* < 0.05; Fig. 4G). TF co-incubation significantly rescued iRPE cells from lipid metabolism imbalance and maintained the detoxification system, as shown by *COX-2* gene expression (*p* < 0.0001 compared to stress condition; Fig. 4F) and GPX4 protein expression (*p* < 0.05 compared with stress; Fig. 4G). The gene expression of acyl-CoA synthetase long chain family member 4 (*ACSL4*), a proferroptotic protein involved in the formation of PUFA, and nuclear receptor coactivator 4 (*NCOA4*), which is involved in the degradation of ferritin by ferritinophagy, was not involved in 4HNE-induced ferroptosis (Fig. 4F).

Taken together, our results demonstrate that lipid peroxidation induces iron overload in iRPE cells and that TF protects these cells from the oxidative, inflammatory, and ferroptotic damage induced by 4HNE, which recapitulates the features of GA.

### TF neutralizes iron-induced toxicity in iRPE cells

**Fig. 5:**
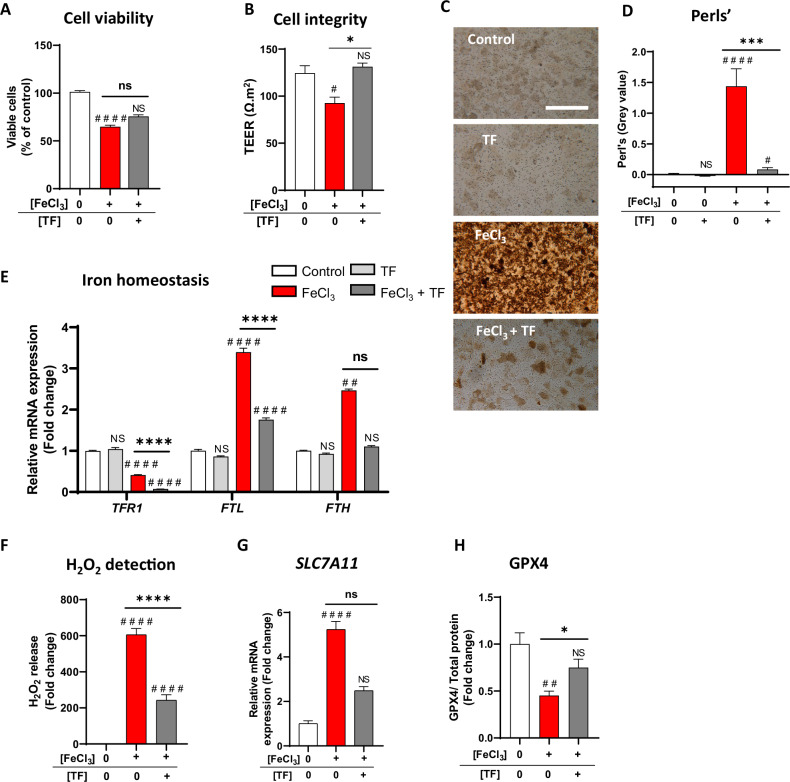
Transferrin neutralizes exogenous iron overload-induced toxicity in RPE cells. iRPE cells were treated for 24 h with 1 mM FeCl_3_ combined with nitrilotriacetate (FeCl_3_NTA) and 20 mg/mL TF, and cell viability (**A**) and integrity (**B**) were preserved (*n* = 4 wells per condition). Iron deposits were revealed by the Perl’s reaction (**C**), and staining intensity quantification (**D**) revealed that intracellular iron accumulation under FeCl_3_ stress strongly decreased with TF co-treatment (**C**: scale bar: 100 µm; **D**: n = 4 wells per condition). **E** Iron stress modulated iron homeostasis, represented by decreased transferrin receptor 1 (*TFR1*) gene expression and increased ferritin light chain (*FTL)* and ferritin heavy chain (*FTH*) gene expression. TF co-treatment significantly increased the decrease in TFR1 and prevented increases in *FTL* and *FTH* (*n* = 6 wells per condition). **F** H_2_O_2_ release from iRPE cells under FeCl_3_ stress conditions was limited when TF was added (*n* = 5–6 wells per condition). **G** The expression of the solute carrier family 7 member 1 (*SLC7A11*) gene, as evaluated by RT‒qPCR, was increased by exogenous iron stress and maintained at the control level with TF co-treatment (*n* = 6 wells per condition). **H** The glutathione peroxidase 4 (GPX4) protein immunoblotting revealed an FeCl_3_-induced decrease, which was significantly limited by TF co-treatment (*n* = 4–6 wells per condition). Untreated cells were used as control. Bars were means ± SEM. One-way ANOVA with post- hoc Bonferroni test (**E**, **F**) or Kruskal‒Wallis test with Dunn’s post-hoc test (**A**, **B**, **D**, **G**, **H**); NS non-significant, #*p* < 0.05, ##*p* < 0.01, ####*p* < 0.0001 compared with the control; ns non-significant, *p < 0.05, ***p < 0.001, ****p < 0.0001 compared with FeCl_3_ stress.

Because lipid peroxidation leads to iron overload in iRPE cells, we further investigated the mechanisms of action of TF on iRPE cells exposed to FeCl_3_ associated with nitrilotriacetate, which forms a stable iron complex in solution. FeCl_3_ induced a dose-dependent reduction in iRPE cell viability and integrity, which reached 50% at 1 mM FeCl_3_ (Supplementary Fig. 4E, F). TF (20 mg/ml) added to iRPE exposed to 1 mM FeCl_3_ for 24 h, setting the ratio at 1:4, partially preserved cell viability (*p* < 0.0001 versus *p* = 0.0729 compared with the control level; Fig. 5A) and maintained cell integrity at the control level (*p* = 0.274 compared to control level) (Fig. 5B). At 24 h, iron loading in iRPE cells (Perls’ staining, *p* < 0.0001; Fig. 5C, D) induced a significant downregulation of transferrin receptor 1 (*TFR1*) (*p* < 0.0001 compared with the control; Fig. 5E) and an upregulation of ferritin light and heavy chain gene expression (*FTL*, *p* < 0.0001 compared with the control; *FTH*, *p* = 0.0082 compared with the control; Fig. 5E). Treatment with TF drastically maintained the intracellular iron level close to the control level (Fig. 5C, D), accentuated the decrease in *TFR1* gene expression (*p* < 0.0001 compared with stress), significantly limited the increase in *FTL* gene expression (*p* < 0.0001 compared with stress) and maintained *FTH* gene expression at control levels (*p* = 0.4270 compared with control) (Fig. 5E). As iron accumulation plays a central role in oxidative stress and the initiation of ferroptosis, we observed a drastic increase in the release of the pro-oxidant H_2_O_2_ (*p* < 0.0001 compared with the control; Fig. 5F), a significant increase in *SLC7A11* gene expression (*p* < 0.01; Fig. 5G) and a significant decrease in the lipid detoxification protein GPX4 (*p* < 0.01; Fig. 5H). TF treatment neutralized iron-induced H_2_O_2_ release by 60% (*p* < 0.0001 compared with stress) and reduced ferroptosis by maintaining *SLC7A11* expression and GPX4 protein expression at control levels (Fig. 5F–H). Interestingly, a similar protective effect of TF was observed in differentiated ARPE-19 cells exposed to FeCl₃, further supporting the relevance of these findings (Supplementary Fig. 6).

These data indicate that exogenous iron overload-induced intracellular iron accumulation, which overwhelmed the iron overload response of RPE cells and initiated ferroptosis. Interestingly, TF, the major cellular iron transporter, neutralizes iron, controls iron homeostasis, and protects RPE cells from iron-induced ferroptosis.

### TF protected iRPE cells from sustained iron overload, which mimics GA features

**Fig. 6:**
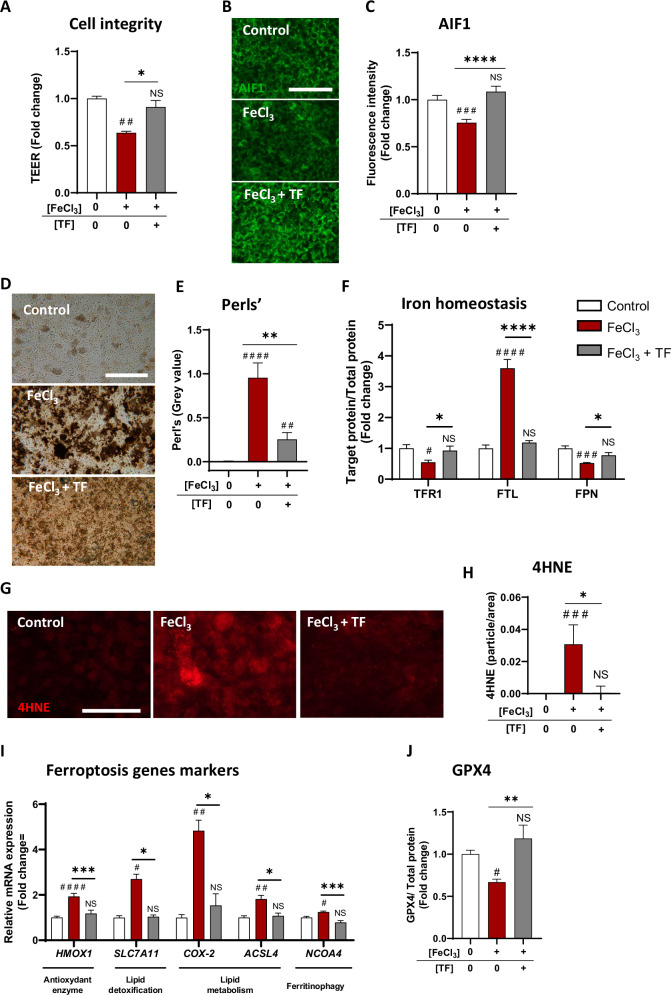
Long-term exogenous iron overload exposure induced ferroptosis, and transferrin preserved iRPE cells from ferroptosis. **A** Sustained iron overload (0.5 mM FeCl_3_NTA for 48 h) induced a significant decrease in iRPE cell integrity, and TF (10 mg/mL) added at 24 h, restored the TEER to the control level (*n* = 4 wells per condition). Mitochondrial viability was quantified via Apoptotic-induced factor 1 (AIF1) immunofluorescence labeling (**B**), which revealed a decrease in AIF1 staining after 48 h of FeCl_3_ exposure (**C**). TF treatment preserved AIF1 staining. (*n* = 4 wells per condition). **D**, **E** Perl’s reaction revealed intracellular iron accumulation in iRPE cells exposed to FeCl_3_ for 48 h. TF added at the half-time of iron exposure decreased iron accumulation (*n* = 4 wells per condition). **F** Transferrin receptor 1 (TFR1), ferritin light chain (*FTL),* and ferroportin (FPN) immunoblotting revealed an iron homeostasis perturbation by sustained iron overload in iRPE cells. The TF treatment restored the protein level to the control level (*n* = 5–6 wells per condition). Sustained iron overload-induced lipid peroxidation, as evaluated by 4HNE immunofluorescence labeling (**G**) and quantified (**H**) after 48 h. TF treatment significantly reduced 4HNE staining compared to FeCl_3_ treatment (*n* = 4 wells per condition). **I** Sustained iron overload in iRPE cells increased the expression of the ferroptosis-related genes heme oxygenase 1 (*HMOX1*), solute carrier family 7 member 1 (*SLC7A11*), cyclooxygenase 2 (*COX-2*), acyl-CoA synthetase long chain family member 4 *(ACSL4*), and nuclear receptor coactivator 4 (*NCOA4*). TF treatment prevented the expression of all the genes (*n* = 6 wells per condition). **J** The glutathione peroxidase 4 (GPX4) protein levels, as quantified by immunoblotting was decreased by sustained iron overload but restored by TF treatment (*n* = 5‒6 wells per condition). Untreated cells were used as control. Bars were means ± SEM. One-way ANOVA with post-hoc Bonferroni test (**F**, **I**, **J**) or Kruskal‒Wallis test with Dunn’s post-hoc test (**A**, **C**, **E**, **H**); NS non-significant, #*p* < 0.05, ##*p* < 0.01, ###*p* < 0.001, ####*p* < 0.0001 compared with the control; ns non-significant, *p < 0.05, **p < 0.01, ***p < 0.001, ****p < 0.0001 compared with FeCl_3_. Scale bar: 100 µm.

Iron accumulation is observed in the retina of AMD patients, and our clinical results suggest that an iron-TF imbalance favors iron overload toxic situation in GA eyes, which still have progression threat. To further analyze the curative potential of TF in a model similar to that of GA, we established a prolonged iron overload model in iRPE cells. Sustained exposure to 0.5 mM FeCl_3_ induced a moderate loss of nearly 20% in iRPE cell viability over 24 h, a sustained alteration in cell integrity at 48 h, and continuous accumulation of intracellular iron at 48 h (Supplementary Fig. 4E, F and Supplementary Fig. 7A–D). Even when added after 24 h of iron exposure, TF (10 mg/mL, 1:4 ratio) was able to restore iRPE cell integrity to the control level (*p* < 0.5; Fig. 6A) and to decrease the mitochondrial dysfunction witnessed by AIF1 immunofluorescence at 48 h (*p* < 0.0001; Fig. 6B, C). TF also protected iRPE cells from intracellular iron accumulation (*p* < 0.0001; Fig. 6D, E), even under prolonged iron overload conditions. In iRPE cells sustained exposed to iron, the expression pattern of iron homeostasis-related proteins confirmed a response to iron overload by a decrease in TFR1 and ferroportin (FPN) expression (*p* < 0.05 compared with the control) and a drastic increase in FTL expression (*p* < 0.0001 compared with the control) (Fig. 6F). TF normalized the expression of TFR1, FTL, and FPN to the level of the control condition (*p* < 0.05, *p* < 0.0001, *p* < 0.05, respectively**;** Fig. 6F).

As it was observed after 24 h of iron exposure, prolonged iron overload activated ferroptosis processes. Forty-eight hours of FeCl_3_ exposure induced lipid peroxidation characterized by an increase in 4HNE staining (*p* = 0.0003 compared to stress; Fig. 6G, H). The expression of *HMOX1 (p* < 0.0001)*, SLC7A11* (*p* = 0.0200), *GPX4* (*p* = 0.0140), *COX-2* (*p* = 0.0083), *ACSL4* (*p* = 0.0048), and *NCOA4* (*p* = 0.0289) significantly increased compared with the control (Fig. 6I), and a decrease in the GPX4 protein level was observed (*p* = 0.0432; Fig. 6J). TF treatment significantly protected iRPE cells from ferroptosis, as evidenced by decreased 4HNE staining (*p* < 0.05) and decreased expression of the *HMOX1* (*p* < 0.001), *SLC7A11* (*p* = 0.0373), *COX-2* (*p* = 0.0171), *ACSL4* (*p* = 0.0275), and *NCOA4* (*p* = 0.0003) genes and GPX4 protein levels (*p* < 0.01) compared with the control (Fig. 6G–J).

**Fig. 7:**
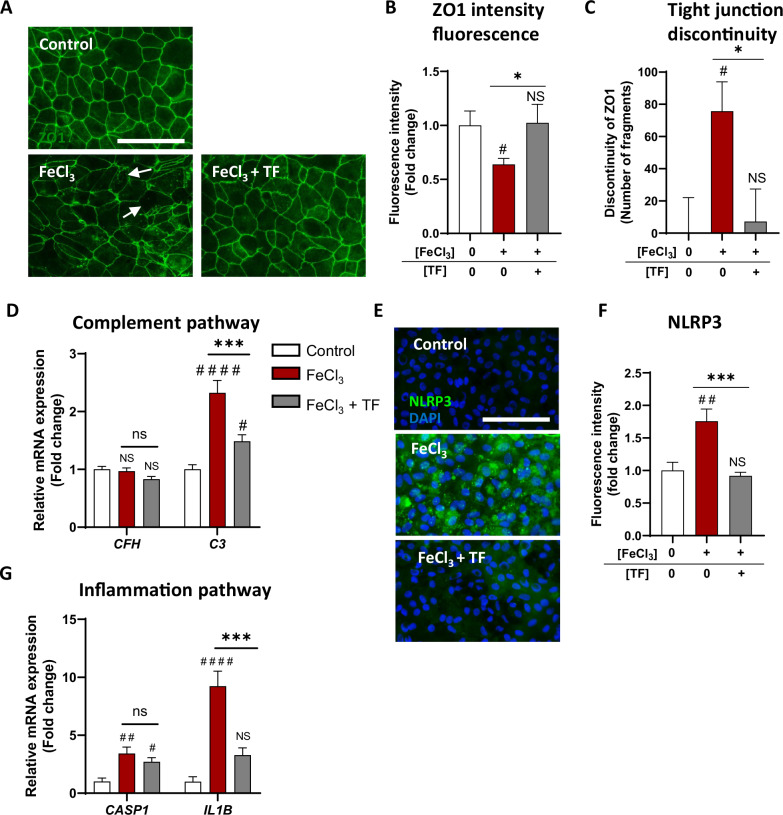
Transferrin preserves iRPE cells from AMD features induced by sustained iron exposure. **A**–**C** Tight junctions were revealed by zonula occludens 1 (ZO1) immunofluorescence labeling (**A**). The staining intensity was measured (**B**), and the number of fragments revealing discontinuity of junctions (**C**) was quantified. A 48-h FeCl_3_ exposure (0.5 mM FeCl_3_NTA) induced a decrease in ZO1 staining intensity (**B**) and an increase of discontinuities (**C**), which were prevented by TF treatment (10 mg/mL) (**A**: scale bar: 100 µm; arrows indicate ZO1 junction discontinuity; **B**, **C**: n = 4 wells per condition). **D** Sustained iron overload in iRPE cells increased *C3* gene expression, which was preserved by TF treatment (*n* = 6 wells per condition). **E**, **F** NLRP3 immunofluorescence labeling was used to assess inflammasome activation in iRPE cells. TF treatment significantly restored the intensity of NOD-like receptor family pyrin domain-containing (NLRP3) staining to the control level (**E**: Scale bar: 100 µm; **F**: n = 4 wells per condition). **G** Sustained iron overload in iRPE cells induced a significant increase in *Interleukin 1 beta* (*IL1B)* gene and *Caspase 1* (*CAS1)* gene expression, as evaluated by RT‒qPCR. TF treatment reduced *IL1B* and *CAS1* gene expression (*n* = 6 wells per condition). Untreated cells were used as control. Bars were means ± SEM. One-way ANOVA with post- hoc Bonferroni test (**D**, **G**) or Kruskal‒Wallis test with Dunn’s post- hoc test (**B**, **C**, **F**); NS non-significant, #*p* < 0.05, ##*p* < 0.01, ####*p* < 0.0001 compared with the control; ns non-significant, *p < 0.05, ***p < 0.001 compared with FeCl_3_ stress.

We then examined AMD features, which characterize the extent of iron-mediated RPE damage. Tight junctions, which are essential for the integrity of the oBRB, were assessed by ZO1 immunofluorescence in iRPE cells. Prolonged iron overload decreased ZO1 staining (*p* = 0.0464; Fig. 7A, B) and increased the number of tight junction discontinuities (*p* = 0.0126; Fig. 7A, C; arrows), and both effects were rescued by TF treatment (*p* = 0.0373; Fig. 7B; *p* = 0.0247; Fig. 7C). The complement pathway activated by prolonged iron overload (high *C3* expression (*p* < 0.0001)) was significantly reduced by TF treatment (*p* = 0.0008 compared with that under stress conditions) (Fig. 7D). Similarly, the inflammasome pathway was activated by prolonged iron overload in iRPE cells, as highlighted by increased NLRP3 protein levels (*p* = 0.0012 compared with those in control cells; Fig. 7E, F) and higher pro-inflammatory gene expression of *CASP1* and *IL1B* (*p* = 0.0012 and *p* < 0.0001, respectively, than in the control condition; Fig. 7G), was decreased by TF treatment. NRLP3 staining and *IL1B* gene expression returned to control levels (*p* = 0.0006 and 0.0004, respectively, compared with those under stress conditions) (Fig. 7E–G). Like 4HNE intoxication, prolonged iron overload mediates RPE damage characteristic of GA. By controlling iron homeostasis, TF protects RPE cells from iron-induced toxicity.

## Discussion

AMD is a complex and multifactorial disease associated with a decline in antioxidant systems, chronic and silent inflammation, and structural changes in the oBRB that contribute to RPE loss and photoreceptor degeneration [47]. Iron plays a central role in these processes, primarily through the Fenton/Haber-Weiss reaction, which generates hydroxyl radicals that drive cholesterol oxidation and lipid peroxidation [48]. The resulting accumulation of 7-ketocholesterol (7-KC), a toxic oxysterol enriched in drusen, contributes to oxidative stress, inflammation, and RPE degeneration in AMD. Iron accumulation and lipid peroxidation are also key features of ferroptosis, a regulated form of cell death increasingly recognized in AMD pathology [9, 49]. There is evidence in humans that iron accumulates in the RPE with age and in drusen and Bruch’s membrane in early dry AMD, where chelatable iron is detected [19]. In AH, increased iron levels have been reported in 12 eyes with nonexudative AMD [20]; however, no studies have investigated the status of iron homeostasis across different stages of nonexudative AMD. In the present study, we measured iron and TF levels in AH collected during cataract surgery from control subjects and patients with AMD, stratified following the recent CAM classification in drusen without any signs of atrophy, iRORA, and cRORA depending on the extension of the RPE and outer retinal atrophy based on SD-OCT [33–35]. Our findings confirm elevated iron levels in the AH of AMD patients [20], with significantly higher iron concentrations specifically in iRORA samples than in controls. In these patients, TF levels were similar to those of controls, leading to a substantially higher TF saturation rate. This saturation level mirrors those found in the plasma of patients with iron overload diseases such as hemochromatosis [50], supporting a potentially toxic iron overload state in the iRORA. Importantly, the iron levels measured in the AH may represent only a fraction of the true exposure. A comparison of the iron and TF levels in the aqueous and vitreous humor from control subjects revealed that the iron concentrations in the vitreous humor were ~10 times higher, whereas the TF levels remained similar. This results in TF saturation nearing 90–100% in the vitreous, indicating a poor iron buffering capacity of the vitreous. These findings also suggest that the iron levels detected in the AH of AMD patients likely underestimate the actual exposure of the retina. Interestingly, we found no significant differences in iron levels or iron-TF balance between cRORA patients and controls. In late-stage GA, particularly in the very advanced cRORA eyes included in our study, the lower iron levels compared with early-stage GA (iRORA) might be attributed to the extensive loss of iron-rich cells such as Müller cells, RPE, and photoreceptors, resulting in reduced iron storage or increased clearance. Additionally, decreased metabolic activity due to the extensive loss of photoreceptors and RPE cells in advanced GA may reduce the retina’s demand for iron and lower oxidative stress, contributing to reduced iron levels in AH. Structural changes in the oBRB as the disease progresses may alter iron dynamics into and out of the retina, leading to redistribution or clearance of iron from the retina. Compensatory changes in iron-regulating proteins could also increase iron sequestration into storage forms, making iron less detectable in AH. Larger studies with a greater number of patients in each subgroup, possibly stratified by GA lesion size, are needed to strengthen these findings and determine whether the iron‒TF balance in AH could serve as a biomarker for GA progression.

The role of iron in AMD has also been evidenced in various animal models of iron overload that mimic features of AMD [51, 52]. Although iRPE cells derived from pluripotent stem cells do not recapitulate the complex microenvironment of the outer retina, they provide a suitable model for studying AMD mechanisms. We chose to evaluate the effects of 4HNE because its pathogenic role in age-related diseases is well established [40]. Other cellular lipids also undergo peroxidation, such as cholesterol, which is even more susceptible to oxidation than polyunsaturated fatty acids. This leads to the early formation of 7-KC, which has been shown to accumulate in the RPE/ choroid and has been identified in drusen in both primates and in humans [53]. In future studies, it will be of interest to compare the effects of 4HNE and 7-KC, as well as the protective effects of TF against these two types of oxidative stressors. We showed that 4HNE leads to iron overload in iRPE cells. This finding links 4HNE to ferroptosis, as evidenced by lipid peroxidation, iron accumulation, nuclear translocation of NRF2, decreased GPX4 protein levels, and increased expression of ferroptotic markers, confirming that 4HNE acts as an inducer of ferroptosis [54]. Additionally, 4HNE or iron (FeCl_3_) exposure caused mitochondrial dysfunction, oxidative stress, inflammasome and complement pathway activation, loss of RPE barrier integrity, and iRPE cell death, which are described in GA. Our results align with those of in vivo iron overload models [55–59] and validate the role of lipid peroxidation and iron overload in activating mechanisms involved in GA. While no model fully recapitulates dry AMD, these two acute in vitro models effectively capture several key features observed in the RPE during the progression of AMD, thereby establishing a relevant platform for evaluating potential therapies for AMD and GA.

Iron chelation is gaining recognition as a promising therapeutic option for AMD/GA, given the significant role of ferroptosis in AMD cell death [9, 49, 60]. Natural polyphenols with iron-chelating properties, such as resveratrol, have shown promising potential in the context of AMD [61]. However, their oral administration may compromise their iron-binding capacity due to metabolic transformations in the gastrointestinal tract and competition for iron by the gut microbiota [62]. This raises important questions about whether their iron-binding sites remain functionally available and, consequently, to what extent iron chelation contributes to their protective effects in the retina. For the treatment of retinal diseases, local administration of iron chelators, especially transferrin (TF), is preferable, as it enables targeted action at sites of iron accumulation without altering systemic iron homeostasis. While chemical iron chelators have been tested in preclinical models [8], they pose risks of retinal toxicity due to the uncontrollable chelation of intracellular iron stores necessary for normal retinal functions [63]. In contrast, TF, as an endogenous regulator of iron homeostasis, mitigates this risk and exhibits neuroprotective activities through both iron-dependent and iron-independent mechanisms [1, 30]. We show herein that TF not only regulates iron import and export to cells, thus reducing intracellular iron accumulation, but also promotes the egress of excess iron from the RPE when added after iron intoxication, corroborating observations from a hemochromatosis animal model [31]. Overall, TF restores iron homeostasis, as demonstrated by the normalization of the expression of genes encoding key iron-regulating proteins, including TFR1, FTL, and FPN. Importantly, as illustrated in graphical abstract, TF inhibits multiple pathogenic mechanisms triggered not only by iron but also by lipid peroxidation in iRPE cells, suggesting that TF is a promising therapeutic option for protecting cells from damage associated with GA.

In conclusion, our findings suggest that TF may protect RPE cells from key pathological mechanisms associated with dry AMD, including oxidative stress, mitochondrial damage, inflammation, complement activation, and ferroptosis due to iron accumulation and lipid peroxidation. Notably, we observed that TF can reduce intracellular iron levels in RPE cells, potentially mitigating iron-induced cellular damage. Consequently, ocular transferrin supplementation may offer a potential therapeutic approach to help mitigate RPE damage and loss, which could contribute to delaying the conversion of AMD to GA or slowing GA progression. The elevated TF saturation observed in the aqueous humor of patients with early-stage GA supports the need for early intervention to maximize the benefits of TF supplementation. In line with these findings, PulseSight Therapeutics (formerly Eyevensys) is advancing the clinical development of PST-611, a transferrin-based non-viral gene therapy for the treatment of dry AMD and GA. This approach aims to achieve sustained intraocular production of TF, thereby addressing chronic iron-mediated damage. A first-in-human clinical trial is expected to commence shortly, pending regulatory approval [64].

## Supplementary information

Supplemental Materials and Methods, and Tables

Supplemental Figures

Uncropped Western Blots

## Data Availability

All the data are available in the main text or the supplementary materials.
