# Supplementary material for: Transferrin is a drug candidate for the treatment of dry age-related macular degeneration (AMD)
Source: Cell Death Dis. 2025 Oct 6;16(1):692. doi: 10.1038/s41419-025-07950-0 (PMC12501284; doi:10.1038/s41419-025-07950-0)
Supplement: Supplementary file 1 — Supplemental Materials and Methods, and Tables [file 41419_2025_7950_MOESM1_ESM.docx]

Transferrin is a drug candidate for the treatment of dry age-related macular degeneration (AMD)

Jenny Youale *et al.*

*Corresponding author. Email:[picardemilie@gmail.com](mailto:picardemilie@gmail.com)

**Supplemental Materials and Methods**

1. **Human ARPE-19 Cell culture**

Human ARPE-19 (ARPE-19) cells were obtained from ATCC (Manassas, VA, USA) and cultured in DMEM/F-12 medium (Thermo Scientific, France) supplemented with 10% fetal bovine serum (FBS; Thermo Scientific), 1% penicillin-streptomycin (Thermo Scientific), and 0.1% fungizone (Thermo Scientific). Cells from passages 5 to 17 were used for all experiments. For stress condition experiments, cells were cultured in serum-deprived DMEM/F-12 medium containing 1% FBS, 1% penicillin-streptomycin, and 0.1% fungizone. All cultures were maintained at 37 °C in a humidified incubator with 5% CO₂. For differentiation, ARPE-19 cells were seeded in 6- or 48-well plates and maintained for 10 weeks in serum-deprived DMEM/F-12 medium (1% FBS, 1% penicillin-streptomycin, 0.1% fungizone) to promote spontaneous differentiation into mature ARPE-19 cells as described by Dunn KC et al.(1) (Dunn KC, Aotaki-Keen AE, Putkey FR, and Hjelmeland LM 1996. ARPE-19, A Human Retinal Pigment Epithelial Cell Line with Differentiated Properties. Exp. Eye Res. 62:155160).

1. **Stress induction and treatment**

Differentiated ARPE-19 (ARPE-19) cells were exposed to either 4-hydroxy-2-nonenal (4HNE; Merck, Saint Quentin Fallavier, France) at 100 µM or FeCl₃-nitrilotriacetate (FeCl₃NTA; Merck) at 100 µM for 24 hours to induce oxidative stress. In rescue conditions, human apo-transferrin (TF; Merck) was co-administered with the stress inducer at a final concentration of 5 mg/mL. Untreated ARPE-19 cells were used as negative controls. All experiments were conducted using at least four independent wells per condition, and cell assignment was performed in a blinded manner.

**Reference:**

1. Dunn KC, Aotaki-Keen AE, Putkey FR, Hjelmeland LM. ARPE-19, a human retinal pigment epithelial cell line with differentiated properties. Exp Eye Res. févr 1996;62(2):155‑69.

**Supplemental Tables**

| Pathway | Gene | mRNA | Ref |
| --- | --- | --- | --- |
| Iron homeostasis | *FTH* | NM_002032 | QT00072681 |
|  | *FTL* | NM_000146 | QT00055860 |
|  | *HEPC* | NM_021175 | QT00200522 |
|  | *TFR1* | NM_001128148 | QT00094850 |
| Ferroptosis | *HMOX1* | NM_002133 | QT00092645 |
|  | *COX-2* (*PTGS2*) | NM_000963 | QT00040586 |
| Inflammation | *CAS-1* | NM_033292 | QT00001568 |
|  | *C3* | NM_000064 | QT00089698 |
|  | *CFH* | NM_000186 | QT00001624 |
|  | *IL1β* | NM_000576 | QT00021385 |

Supplementary Table 1. Primers (10X QuantiTect Primer Assay, Qiagen)

| Pathway | Gene |  | Sequence (5’-3’) |
| --- | --- | --- | --- |
| Ferroptosis | *ACSL4* | Foward | ATAAAGCAGAGTACCCTGAAG |
|  |  | Reverse | CAAGTTTTCTGGGTTAGATCC |
|  | *NCOA4* | Foward | AACAGTCAGACTTCTTCCAG |
|  |  | Reverse | TAGTAGTGGAATGGCTGTTAC |
|  | *SLC7A11* | Foward | GGTTATTCTATGTTGCGTCTC |
|  |  | Reverse | AATAACAGCTGGTAGAGGAG |

Supplementary Table 2. Primers (Merck)
