# Supplementary material for: Transferrin is a drug candidate for the treatment of dry age-related macular degeneration (AMD)
Source: Cell Death Dis. 2025 Oct 6;16(1):692. doi: 10.1038/s41419-025-07950-0 (PMC12501284; doi:10.1038/s41419-025-07950-0)
Supplement: Supplementary file 2 — Supplemental Figures [file 41419_2025_7950_MOESM2_ESM.docx]

Transferrin is a drug candidate for the treatment of dry age-related macular degeneration (AMD)

Jenny Youale *et al.*

*Corresponding author. Email:[picardemilie@gmail.com](mailto:picardemilie@gmail.com)

**Supplemental Figures**


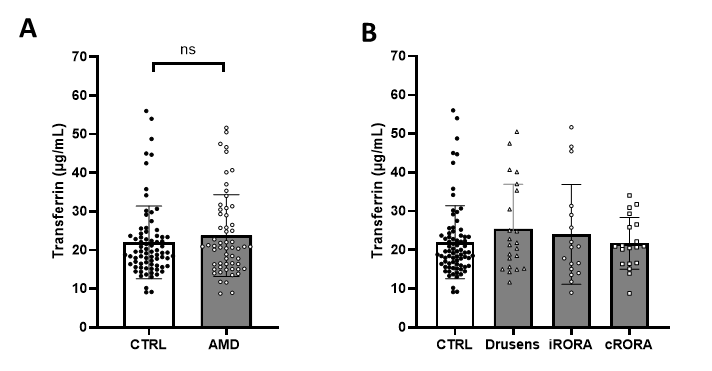


**Supplementary** **Figure 1. Transferrin concentration in atrophic geography (GA) age-related macular degeneration (AMD) patients**

Transferrin (TF) concentration in aqueous humor from control (40 males and 37 females) and GA AMD patients (37 males and 21 females) (**A)** and in different AMD subtypes classified according to the Classification Atrophy Meeting (**B**). Bars represented means ± SD. (A) Mann‒Whitney test, (B) Kruskal‒Wallis’ test; Dunn’s post hoc test. ns: non-significant.


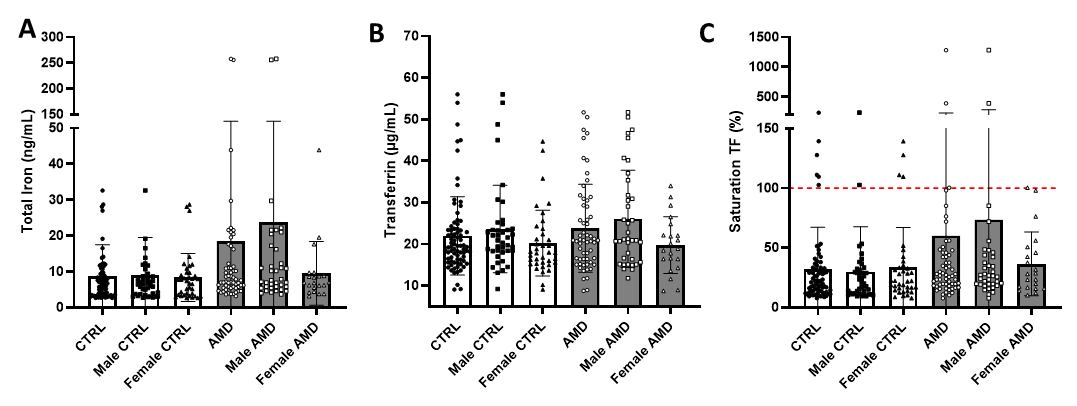


**Supplementary Figure 2. Total iron and transferrin saturation in atrophic geography (GA) age-related macular degeneration (AMD) patients depending on sex.**

The total iron level (**A**), transferrin (TF) concentration (**B**) and TF saturation (**C**) in aqueous humor from control (40 males and 37 females) and GA AMD patients (37 males and 21 females) did not significantly differ according to sex. The bars represent the means ± SDs. Kruskal‒Wallis’ test; Dunn’s post hoc test.


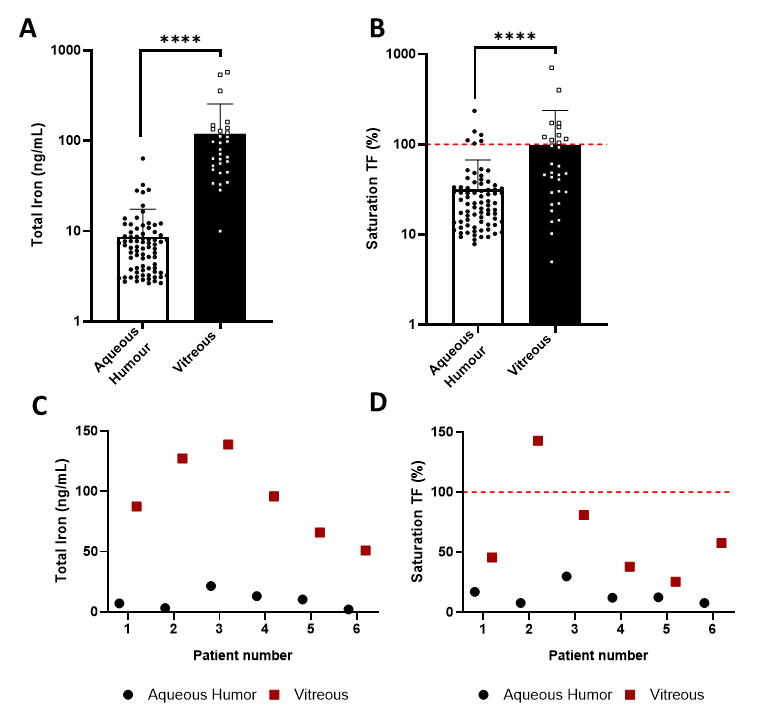


**Supplementary Figure 3.** **Comparison of iron status in aqueous humor and vitreous from control eyes.**

(**A-B**) Total iron (A) and transferrin (TF) saturation (B) were significantly higher in vitreous (n= 30) than in aqueous humor (n= 77) from control patients. Bars represented means ± SD. Mann-Whitney’s test. **** *p* < 0.0001. (**C-D**) Total iron (C) and TF saturation (D) quantified in aqueous humor and vitreous samples collected at the same time from control patients.


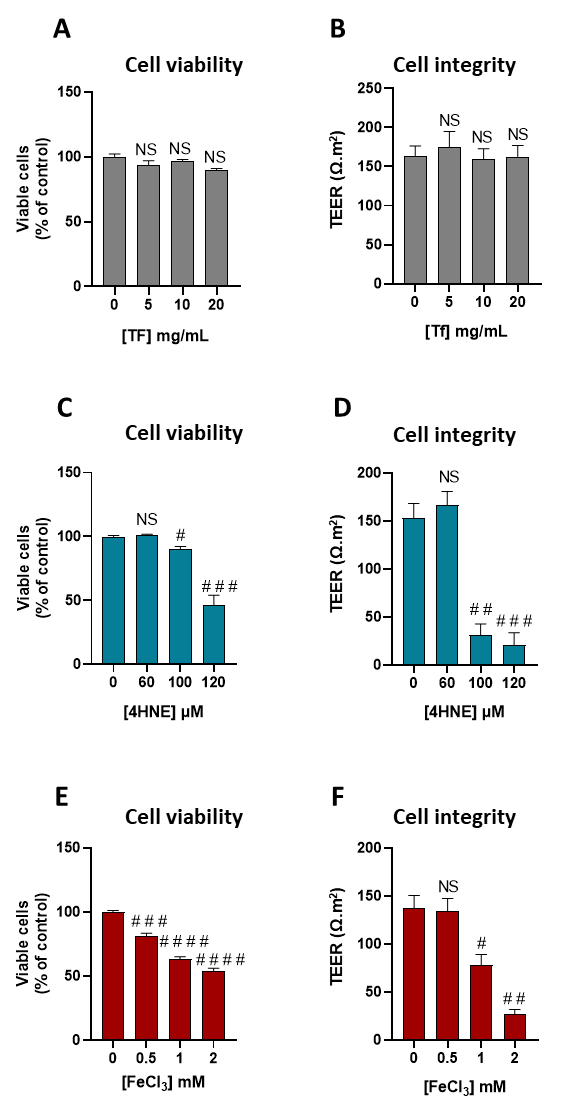


**Supplementary Figure 4. Transferrin safety and toxicological screening of the effects of 4HNE and FeCl_3_ on iRPE**

(**A-B**) To evaluate transferrin (TF) safety, human induced pluripotent stem cell (iPSC)-derived RPE (iRPE) cells were exposed to 5, 10 or 20 mg/mL TF for 24 hours. No significant difference in (**A**) cell viability (n = 3 wells per condition) or (**B**) iRPE cell transepithelial electrical resistance (TEER) (n = 7 wells per condition) was observed among the different conditions tested. (C**-F**) For toxicological screening, iRPE cells were exposed to 60, 100 or 120 µM 4-hydroxy-2-nonenal (4HNE) or 0.5, 1 or 2 mM FeCl_3_, respectively, for 24 hours. 4HNE induced a significant concentration-dependent decrease (**C**) in iRPE cell viability and (**D**) in iRPE TEER from 100 µM onward (n = 3–7 wells per condition). FeCl_3_ induced a significant concentration-dependent decrease in iRPE cell viability (**E**) and TEER (**F**) from 0.5 mM onward (n = 8–12 wells per condition). Untreated cells were used as control. Bars were means ± SEM. Kruskal‒Wallis’ test; Dunn’s post hoc test; NS non-significant, # p < 0.05, ## p < 0.01, ### p < 0.001, #### p < 0.0001 compared with the control.


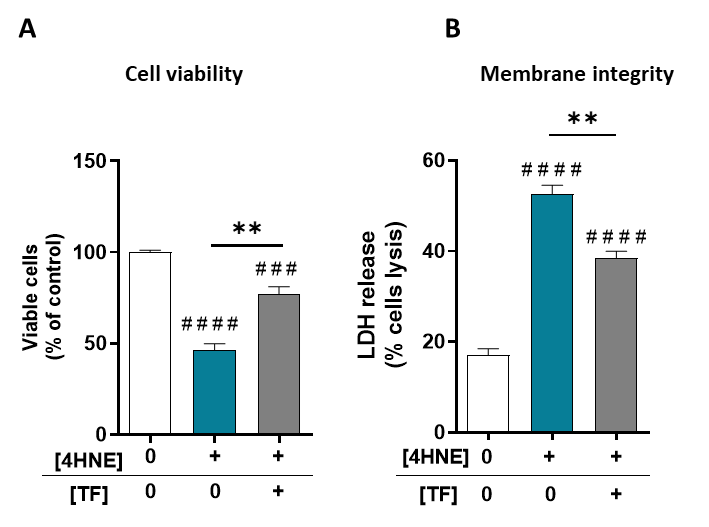


**Supplementary Figure 5**: **Transferrin preserved APRE-19 cells from 4HNE-induced toxicity**

(**A**) A 24-hour incubation with 100 µM 4-hydroxy-2-nonenenal (4HNE) significantly reduced differentiated human ARPE-19 cell viability compared with that of the control. Co-treatment with 5 mg/mL TF significantly protected ARPE-19 cells from 4HNE-induced toxicity (n = 20-21 wells per condition). (**B**) Under 4HNE stress, membrane integrity, monitored by the release of lactate dehydrogenase (LDH), was lost, and TF co-treatment significantly prevented this loss (n = 25-29 wells per condition). Untreated cells were used as control. Bars were means ± SEM. Kruskal‒Wallis’ test with Dunn’s post-hoc test; ### *p* < 0.001, #### *p* < 0.0001 compared with the control; ** *p* < 0.01 compared with the 4HNE stress.

 
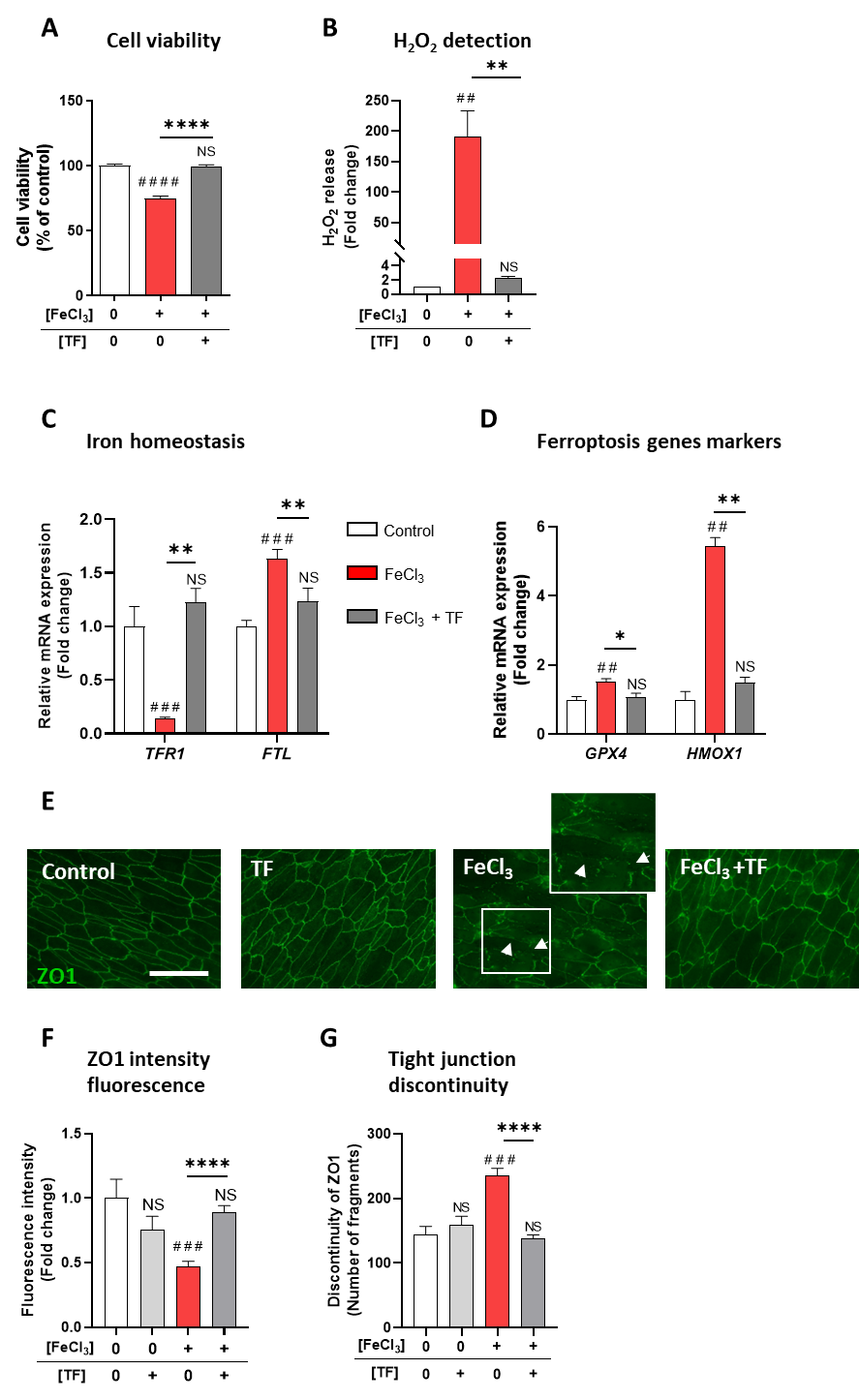


**Supplementary Figure 6**: **Transferrin neutralizes exogenous iron overload-induced toxicity in dARPE-19 cells**

(**A**) Differentiated ARPE-19 cells were treated for 24 hours with 100 µM FeCl_3_ combined with nitrilotriacetate (FeCl_3_NTA) and 5 mg/mL TF, and cell viability was preserved (n = 18 wells per condition). (**B**) H_2_O_2_ release from ARPE-19 cells under FeCl_3_ stress conditions was limited when TF was added (n = 4–6 wells per condition). (**C**) Iron stress modulated iron homeostasis, represented by decreased transferrin receptor 1 (*TFR1*) gene expression and increased ferritin light chain (*FTL*) gene expression. TF co-treatment significantly prevented the decrease in TFR1 and prevented increases in *FTL* (n = 6 wells per condition). (**D**) Iron stress in ARPE-19 cells increased the expression of the ferroptosis-related genes *GPX4* and *HMOX1.* TF treatment prevented the expression of both genes (n = 6 wells per condition). (**E**) Tight junctions were revealed by zonula occludens 1 (ZO1) immunofluorescence labeling. The staining intensity was measured (**F**), and the number of fragments revealing discontinuity of junctions (**G**) was quantified. A 24-hour FeCl_3_ exposure (100 µM FeCl_3_NTA) induced a decrease in ZO1 staining intensity (**F**) and an increase of discontinuities (**G**) which were prevented by TF treatment (5 mg/mL) (scale bar: 100 µm; arrows indicate ZO1 junction discontinuity; F‒G: n = 4 wells per condition). Untreated cells were used as control. Bars were means ± SEM. One way ANOVA with post- hoc Bonferroni’s test (A-C) or Kruskal‒Wallis’ test with Dunn’s post-hoc test (B, D, F-G); NS: non-significant, ## *p* < 0.01, ### *p* < 0.001,  #### *p* < 0.0001 compared with the control; ns non-significant, * *p* < 0.05, ** *p* < 0.01, *** *p* < 0.001, **** *p* < 0.0001 compared with FeCl_3_ stress.

 
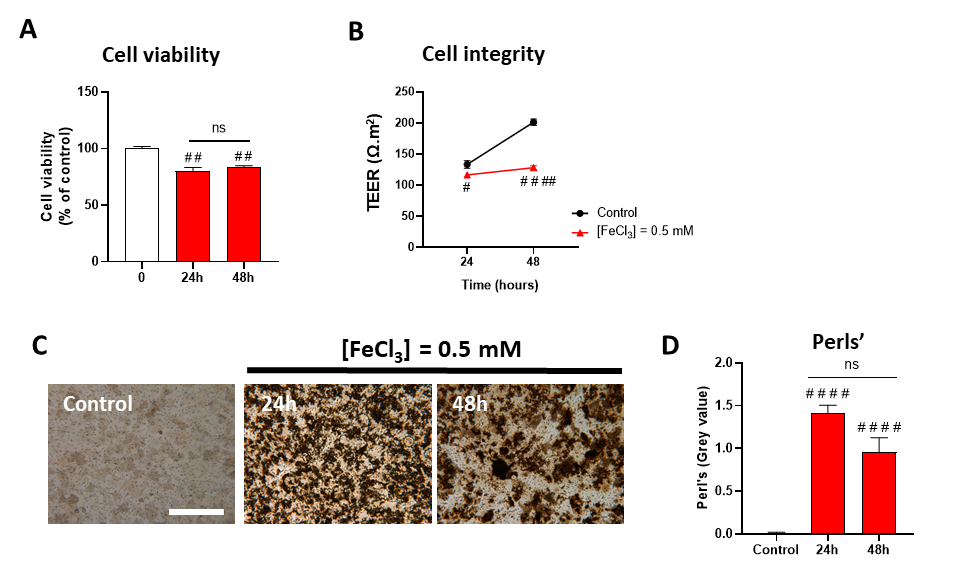


**Supplementary Figure 7. Time course effects of iron exposure**

(**A**) The viability of iRPEs exposed to FeCl_3_ for 24 or 48 hours was significantly lower than that of the control (n = 6–14 wells per condition). (**B**) Cell integrity was significantly reduced from 48 hours of FeCl_3_ exposition compared with the control (n = 3–11 wells per condition). (**C-D**) Perl’s reaction revealed significant intracellular iron accumulation in iRPE cells induced by sustained iron overload (0.5 mM FeCl_3_) compared with that in control cells after 24 hours of exposure (C: scale bar 100 µm; D: n = 4 wells per condition). Untreated cells were used as control. Bars were means ± SEM. Kruskal-Wallis’ test; Dunn’s test; NS non-significant, # *p* < 0.05, ## *p* < 0.01, ### *p* < 0.001, #### *p* < 0.0001 compared with the control.
