# Supplementary material for: Transferrin is a drug candidate for the treatment of dry age-related macular degeneration (AMD)
Source: Cell Death Dis. 2025 Oct 6;16(1):692. doi: 10.1038/s41419-025-07950-0 (PMC12501284; doi:10.1038/s41419-025-07950-0)
Supplement: Supplementary file 3 — Uncropped Western Blots [file 41419_2025_7950_MOESM3_ESM.docx]

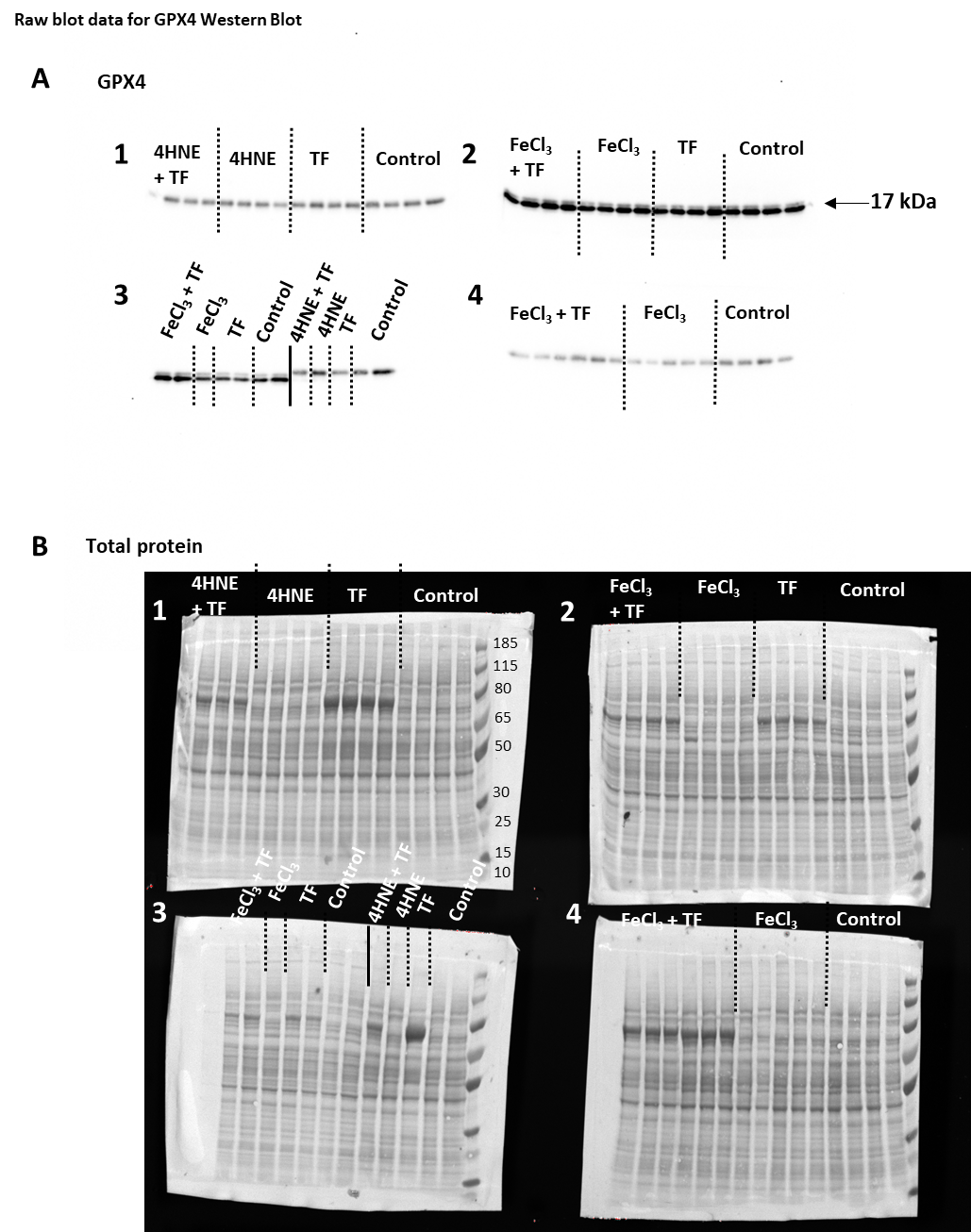


**Legend : Raw blot data for GPX4 Western Blot :** Pictures of the GPX4 immunoblotting (**A**) and total protein staining (**B**). 1: Samples corresponding to Figure 4G (4HNE stress); 2: Samples corresponding to Figure 6J (48 hours iron stress) ; 3: Samples corresponding to Figure 6J (left) and Figure 4G (right); 4: Samples corresponding to Figure 5H (24 hours iron stress)


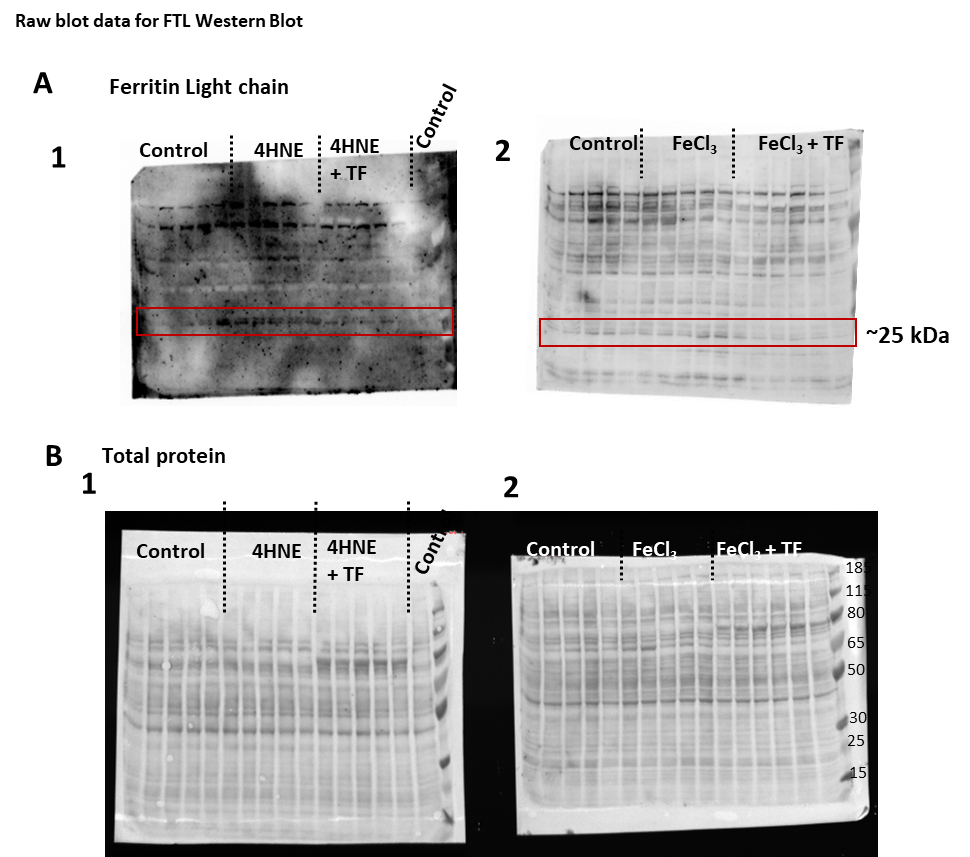


**Legend : Raw blot data for Ferritin Light Chain Western Blot :** Pictures of the FTL immunoblotting (**A**) and total protein staining (**B**). 1: Samples corresponding to Figure 4C (4HNE stress); 2: Samples corresponding to Figure 6F (48 hours iron stress).


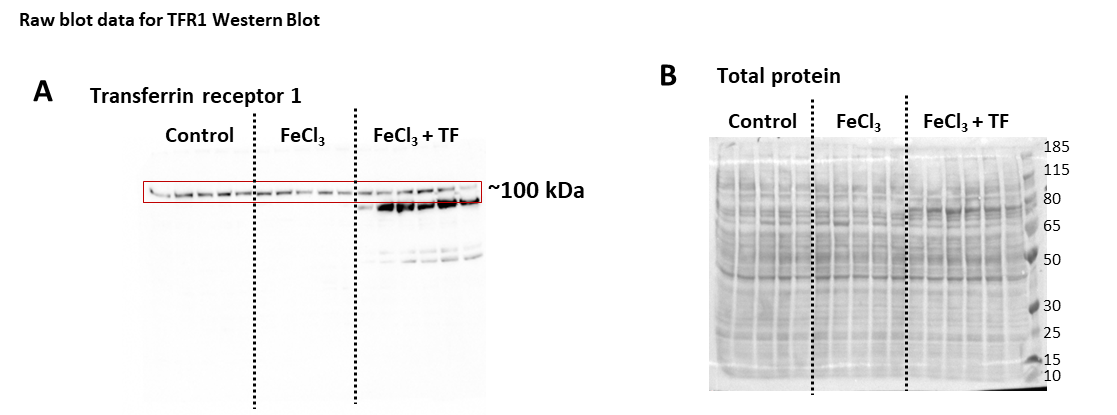


**Legend : Raw blot data for Transferrin Receptor 1 Western Blot :** Pictures of the RTF1 immunoblotting (**A**) and total protein staining (**B**). Samples corresponding to Figure 6F (48 hours iron stress).


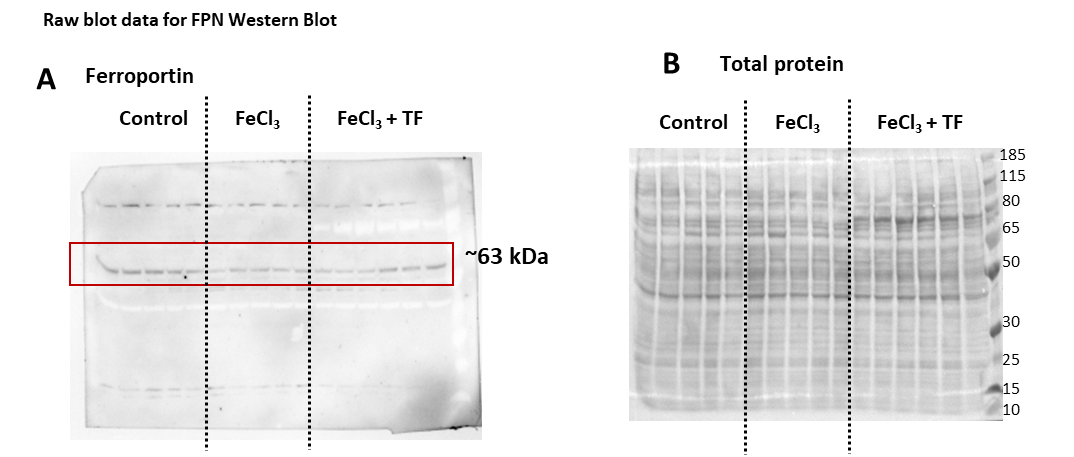


**Legend : Raw blot data for Ferroportin Western Blot :** Pictures of the FPN immunoblotting (**A**) and total protein staining (**B**). Samples corresponding to Figure 6F (48 hours iron stress).
